# Encasing the paramagnetic copper(ii)-ion by the ring-contracted corrin ligand of vitamin B_12_†‡

**DOI:** 10.1039/d5cc02129d

**Published:** 2025-06-09

**Authors:** Christoph Kieninger, Klaus Wurst, Daniel Leitner, Luis P. Peters, Dennis F. Dinu, Markus Wiedemair, Marc-Kevin Zaretzke, Martin Bröring, Stephan Hohloch, Klaus R. Liedl, Bernhard Kräutler

**Affiliations:** a Institute of Organic Chemistry, University of Innsbruck 6020 Innsbruck Austria bernhard.kraeutler@uibk.ac.at; b Center for Molecular Biosciences (CMBI), University of Innsbruck 6020 Innsbruck Austria; c Institute of General, Inorganic & Theoretical Chemistry, University of Innsbruck 6020 Innsbruck Austria; d Institute for Inorganic and Analytical Chemistry, TU Braunschweig 38106 Braunschweig Germany

## Abstract

The d^9^-Cu(ii)-corrin cupribyrate (Cuby) was synthesized in 93% crystalline yield by rapid chelation of Cu^2+^-ions by the metal-free corrin-ligand of vitamin B_12_. Single crystals of the EPR-active Cuby allowed for the first X-ray structure determination of a Cu-corrin. SCF-calculations provided insights complementary to the experimental data of Cuby and indicated an out-of-plane displacement of the reduced d^10^-Cu(i)-ion, consistent with the observed reductive activation of Cuby towards loss of its Cu-center.

## Abstract 2

The encasement of a Cu(ii)-ion by the ring-contracted corrin-ligand of vitamin B_12_ leads to a tightly bound and nearly planar, 4-coordinate d_9_-Cu(ii)-centre, whose unpaired electron occupies a largely metal-located, antibonding d_*x*^2^–*y*^2^_ orbital.

## Graphical abstract

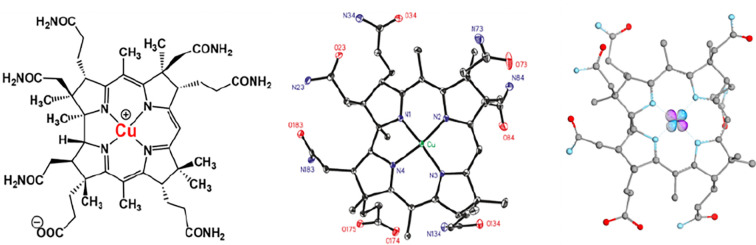

The ring-contracted natural corrin ligand of the B_12_-derivatives is a uniquely skewed, helical environment^1,2^ that binds cobalt-ions very tightly.^3,4^ This biosynthetically costly ligand for cobalt^5,6^ represents a precisely evolved entatic state module,^2^ giving B_12_-cofactors the unique capacity for their exceptional bio-organometallic catalysis.^7–9^ The complementary fundamental question, why cobalt? in B_12_-cofactors,^1,3,9,10^ has generated the long-standing experimental quest for non-cobalt analogues of the B_12_-derivatives,^11,12^ a challenge met by newly developed synthetic approaches.^2,13^ We have, thus, prepared Rh(iii)-,^13–17^ Ni(ii)-^18^ and Zn(ii)-complexes^19^ of natural corrin ligands for studies of their structures and reactivity. Here, we report on cupribyrate (Cuby) (Scheme 1), the Cu(ii)-complex of hydrogenobyric acid (Hby),^2^ including the first Cu-corrin X-ray crystal structure.

**Scheme 1:**
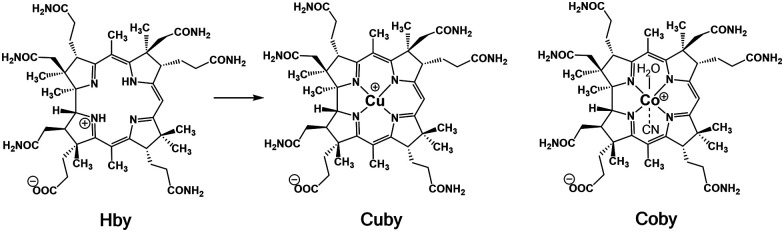
Structure-based outline of the synthesis of cupribyrate (Cuby) from hydrogenobyric acid (Hby) (see the ESI‡) and the structural formula of Co_α_cyano, Co_β_aquo-cobyric acid (Coby).

The complexation of metal-free Hby with Cu(ii)-ions occurred readily at room temperature (RT) in a 0.25 M aqueous solution of Cu(ii)-acetate at pH 6 and was practically quantitative within 90 min (see the ESI‡). It did not require the reported strong heating (‘brief boiling’).^11,20^ Crystallization of the raw Cuby-isolate from water/acetonitrile mixtures furnished Cuby in >93% yield.

The UV/Vis-spectrum (Fig. 1) of an aqueous solution of Cuby exhibits a corrin-type and is comparable to the earlier reported spectra of partially characterized Cu(ii)-corrins.^20,21^ UV/Vis- and CD-spectra of Cuby show remarkably similar features to the corresponding spectra of the Zn(ii)-complex^19^ of Hby, consistent with the dominating role of the corrin chromophore for the spectral signature in the UV- and Vis-range. A HR-ESI mass spectrum of Cuby confirmed the calculated molecular formula of C_45_H_64_CuN_10_O_8_ (see the ESI,‡ Fig. S1).

**Fig. 1:**
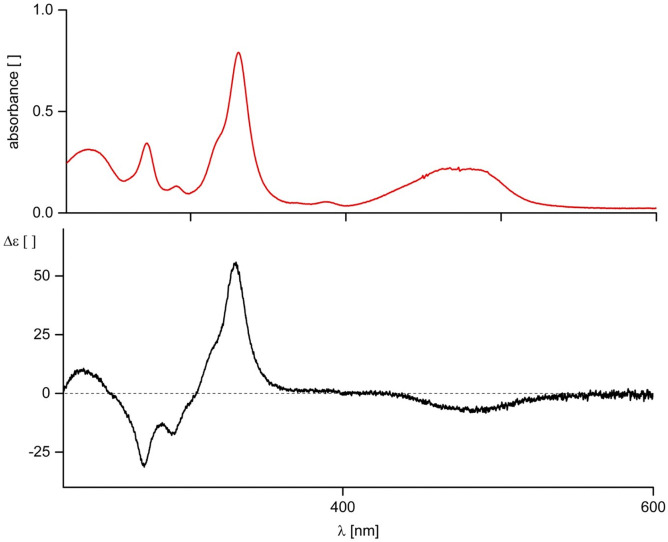
UV/Vis- and CD-spectra of Cuby (19 μM in 10 mM aqueous phosphate pH 7).

Glassy frozen solutions of the paramagnetic Cu(ii)-corrin Cuby in 20% glycerol in H_2_O showed the typical EPR-signature (see Fig. 2, for a spectrum at *T* = 148 K) of a roughly square-planar 4-coordinate Cu(ii)-N_4_-complex with an index^22,23^ g^II^/A^II^ = 98.2 cm, assigning an exceptionally low value to the encasement of the Cu(ii)-ion by the corrin ligand (see the ESI‡ for further details).

**Fig. 2:**
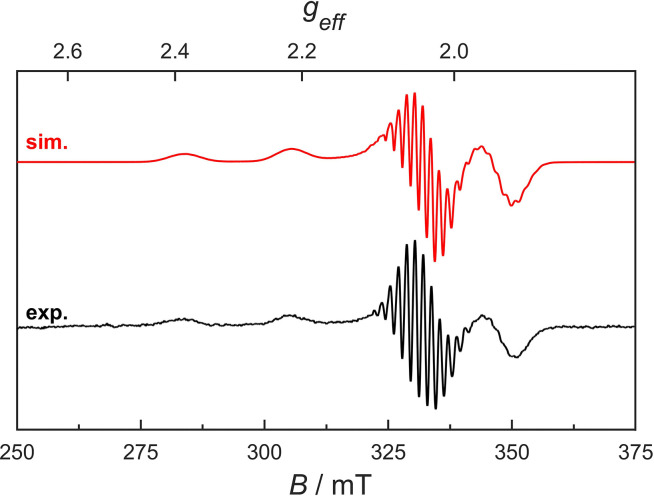
EPR-spectrum of a 1.34 mM frozen solution (at *T* = 148 K) of Cuby in H_2_O:glycerol (4 : 1) and its simulation with key parameters obtained by least square fitting (for details see the ESI,‡ Table S1). The spectra exhibited a significant T-dependence, with a maximum signal intensity of around 200 K and continuous decrease at lower temperatures (see the ESI,‡ Fig. S4).

The neutral cupribyrate Cuby crystallized from an aqueous solution upon addition of acetonitrile. The monoclinic crystals (space group *P*2_1_) contain two Cuby molecules per unit cell, as well as molecules of water and acetonitrile (ordered near the Cuby-carboxylate). The Cu(ii)-center of the Cuby molecule sits only +0.033 Å above the mean plane of the four ‘inner’ corrin N-atoms, which span an unsymmetrical and nearly planar coordination pattern (see Fig. 3), as reflected by the value of the geometry index *τ*_4_ = 0.17.^24^ However, Cuby exhibits a less planar arrangement around its 4-coordinate d^9^ Cu(ii)-center, than in the Ni(ii)-corrin nibyrate (Niby),^18^ which experiences a better fit of its 4-coordinate low-spin d^8^ Ni(ii)-ion (see the ESI,‡ Table S3). The average Cu–N distance in Cuby amounts to 1.91 Å, merely 0.05 Å longer than in Niby, in which the 0.08 Å smaller low spin d^8^-ion Ni(ii)^25^ induced a slight contraction.^18^ In fact, binding of the d^9^ Cu(ii)-ion largely retains the architecture of the coordination hole of the metal-free corrin ligand Hby, expanded by two ‘inner’ protons.^2^ In Cuby, the critical angle parameters corrin-fold^26^ (10.0°) and corrin helicity^2^ (12.4°) are also similar to those of the ligand Hby,^2^ but remarkably larger than in Niby. Likewise, the angle between the planes N1–Cu–N2 and N3–Cu–N4 (roughly 13.6°) relating to the inner coordination-sphere around the Cu(ii)-center (see the ESI,‡ Table S3) is close to the value derived for Hby.^2^ Interestingly, the N1–M–N3 pseudo-diagonal in Cuby was roughly 0.07 Å shorter than the N2–M–N4 counterpart, thus displaying a larger difference of the distances across these pseudo-diagonals than in Niby. This desymmetrization of the corrin core in Cuby also goes along the one observed in Hby and its Zn(ii)-complex,^19^ but is insignificant in the Co(ii)-corrins Co(ii)-cobalamin (Cbl^II^)^27^ and cob(ii)ester^28^ and in typical Co(iii)-corrins, such as coenzyme B_12_ (AdoCbl)^29^ and vitamin B_12_.^30,31^

**Fig. 3:**
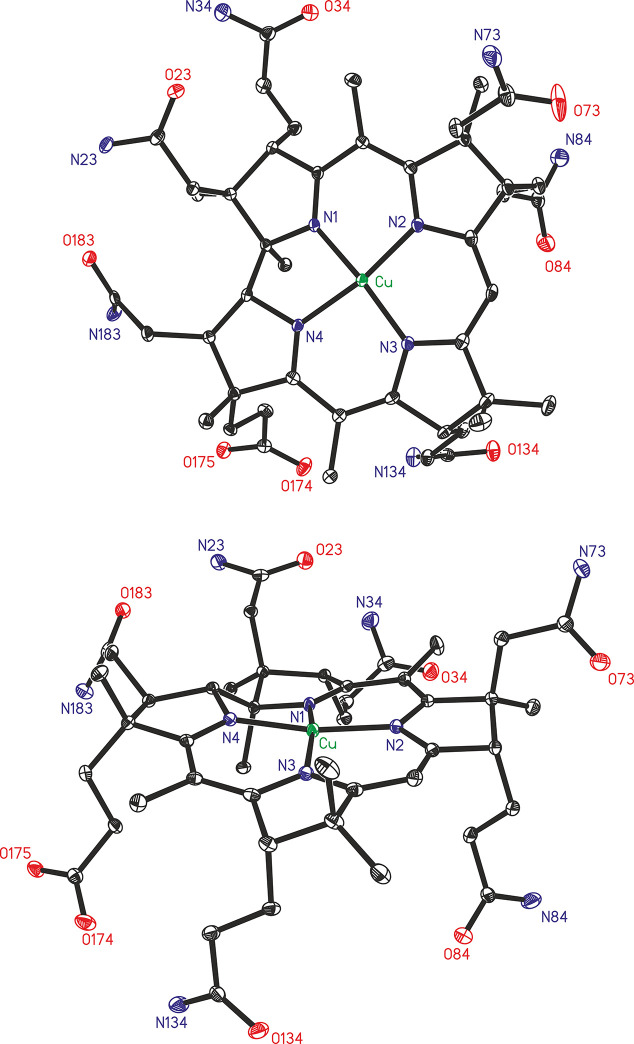
X-ray crystal structure of Cuby in ORTEP-representations. Top: axial view from above (β-side); bottom: approximate in-plane view, revealing the slightly nonplanar 4-fold coordination of the encased Cu(ii)-ion.

Our self-consistent field (SCF) in the gas-phase calculation of cupribyric acid (HCuby+), the cationic carboxylate-protonated form of Cuby, used the atomic coordinates of the Cuby crystal structure. In this model, large artefactual electron density contributions of the carboxylate function of Cuby to occupied MOs were lacking, consistent with the experimental absence of such interactions. The so-derived computational insights into the bonding interactions of a d^9^-Cu(ii)-corrin were fully consistent with separately located calculated frontier molecular orbitals, either as π-type corrin ligand MOs or as the singly occupied d_*x*^2^–*y*^2^_-type orbital on the Cu(ii)-center (see Fig. 4 and ESI‡ Fig. S13). We also tested computed models of cuprobyric acid (Cu(i)by) to shed light on the difficult^32^ one-electron reduction to a d^10^-Cu(i)-corrin (for details, see the ESI,‡ Fig. S14). The calculations suggest a large upper axial out of plane movement of the Cu(i)-ion, comparable to the position of the rather weakly bound Zn(ii)-center in zincobyric acid (Znby).^19^ Indeed, the iso-electronic nature of the closed shell d^10^-ions Cu(i) and Zn(ii) suggested the likelihood of the complete removal of a Cu(i)-ion from reduced Cuby in a weakly acidic aqueous medium. In an exploratory experiment, Cuby was treated with Zn-powder in an aqueous NH_4_Cl solution, leading to the effective replacement of the Cu-center of Cuby by Zn(ii), furnishing Znby,^19^ and its tentatively (by mass- and UV/Vis-spectroscopy) characterized dihydro-form H_2_-Znby, an unprecedented ring-reduced yellow corrinoid^33,34^ (see ESI,‡ Scheme S1). We ascribe the observed formation of Znby from Cuby to a transient generation of an exchange-labile d^10^-Cu(i)-center by the Zn-reduction, thus strategically circumventing Eschenmoser's postulate that a B-type transition metal could not be removed without destruction of the corrin-ligand.^35^

**Fig. 4:**
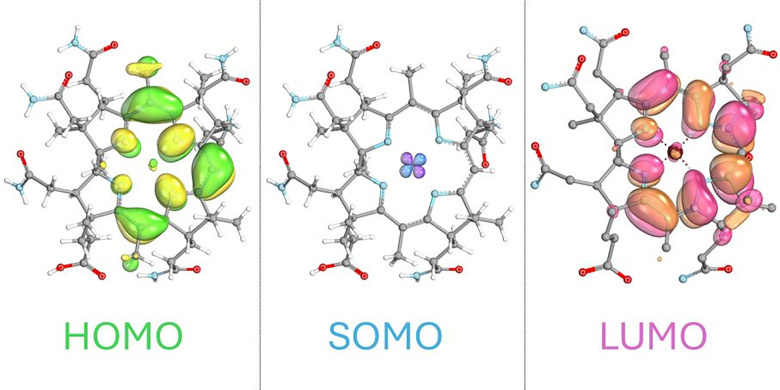
Frontier molecular orbitals (FMOs) of the cupribyric acid cation (HCuby^+^) from the self-consistent field in gas-phase calculations. From left to right: the highest occupied MO (HOMO), a corrin π-type orbital; the d_*x*^2^–*y*^2^_-type Cu(ii)-located singly occupied MO (SOMO); and the lowest unoccupied MO (LUMO), a corrin π-type orbital. Orbitals are seen from the upper side.

The replacement, by copper, of the biologically selected cobalt-center of a corrinoid B_12_-derivative^1,36,37^ erases its fundamental organometallic redox-reactivity.^7^ The single unpaired electron of the paramagnetic Cuby does not contribute any (cobalt-mimetic) radical reactivity, but is ‘buried’ in a d_*x*^2^–*y*^2^_ orbital of its d^9^ Cu(ii)-center. Consistent with the EPR-spectral fingerprint of Cuby and its large ^14^N-hfcs with the four inner corrin N-atoms, in particular, the unpaired spin is located in a d_*x*^2^–*y*^2^_ orbital of an effectively antibonding type with respect to the coordinating corrin N-atoms (Fig. 4). Compared to the d^8^ Ni(ii)-ion in Niby, the Cu(ii)–N bonds in Cuby are, indeed, longer. Copper complexes of the superficially similar corroles represent a remarkably more complex situation:^38,39^ there ‘non-innocence’ of the corrole ligand is caused by its extended π-system, assisting an intramolecular electron-shift and stabilizing the copper center in a higher oxidation state.^40^

The chelation of the fluorescent metal-free corrin Hby^2,41^ by Cu(ii)-ions in aqueous solution occurs cleanly at ambient temperature at pH 5. The Cu(ii)-ions chelate **Hby** with a rate *k*^Cu(II)^ = 0.54 ± 0.04 L mol^−1^ min^−1^, remarkably quicker by about 2 × 10^2^ times than the binding of the biologically crucial Co(ii)-ions (*k*^Co(II)^ of about 3 × 10^−3^ L mol^−1^ min^−1^), and five times faster than Zn(ii)-ions (*k*^Zn(II)^ = 0.111 ± 0.002 L mol^−1^ min^−1^, see the ESI‡). The chelation rates of these metal ions follow the trend established with Eschenmoser's model corrin^35^ and with a water-soluble tetra-mesopyridyl-porphyrin.^42^

Obviously, the biological roles of Co^7,8,43^ and Cu^44–47^ do not match. However, the 4-coordinate Cu(ii)-complexes of natural corrin ligands may serve as structural mimics of reduced B_12_-derivatives. In concert with the divergent reactivity profiles of copper- and cobalt-corrins, biologically interesting applications are likely. Cuby is structured similar to the corrin-core of enzyme-activated 4-coordinate Co(ii)-cobamides, first characterized in an ATP:Co(i)-corrinoid adenosyltransferase that generates AdoCbl from 4-coordinate Co(ii)-Cbl.^48^ With their largely inert 4-coordinate d^9^- and d^8^-metal-centers, respectively, Cu(ii)- and Ni(ii)-corrins^18^ may effectively mimic the structures of the highly activated 4-coordinate Co(ii)- and Co(i)-corrins. Indeed, nibalamin (Nibl), the diamagnetic Ni(ii)-analogue of ‘base-off’ Co(ii)-Cbl, was shown to be an effective inhibitor of the corrinoid adenosyltransferase BtuR from *Brucella melitensis*.^18^ The crystal structure of Cuby qualifies Cu(ii)-containing B_12_-derivatives, such as cupribalamin (Cubl), for similar inhibitory effects.

Transition metal analogues of vitamin B_12_ and other cobalamins (Cbls), also classified as metbalamins (Metbls),^49,50^ lack the precise cobalt-dependent reactivity of Cbls^8,43^ and, when mimicking Cbl-structures, may represent genuine antivitamins B_12_.^50,51^ This is the case for rhodibalamins (Rhbls), the Rh(iii)-homologues of Cbls. Surprisingly, their Rh(iii)-center has even been revealed to experience a slightly better fit to the corrin ligand than the naturally selected Co(iii)-ions.^13–15^ Whereas uptake and physiological activity of Metbls with stable 4-coordinate corrin-bound metal centers are still unknown in humans and animals, microorganisms are typically more structure-promiscuous for B_12_-import, satisfying their supply with cobamides by *de novo* biosynthesis^5^ or by partial assembly from salvaged natural corrinoids.^52,53^ As deduced for some Rhbls^13,15^ and for Nibl,^18^ transition metal-based structural mimics of B_12_-cofactors or of corrinoid B_12_-biosynthesis intermediates^17^ may selectively inhibit bacterial growth. As mimics of enzyme-bound Cbl-structures in B_12_-dependent enzymes at intermediate stages of catalysis, Metbls may specifically act as very effective enzyme inhibitors. The Cu(ii)-analogues of natural B_12_-derivatives are, hence, EPR-active candidates for their applications as B_12_-antimetabolites for B_12_-dependent microorganisms, an expansion of the toolbox of Cu-coordinating natural products^47^ as antimicrobial agents.

Synthetic, analytical and spectroscopic work: C. K. and M. W.; crystallography: C. K. and K. W.; theoretical and computational study: L. P. P., D. F. D., and K. R. L.; EPR-spectroscopy – data acquisition, supervision and data curation: D. L., M.-K. Z., M. B., and S. H.; and research conceptualization and conduction and original draft: B. K.; all authors have reviewed and contributed to the final draft.

We are particularly grateful to Evelyne Deery and Martin Warren for a generous supply of hydrogenobyric acid. This work was supported by the Austrian Science Fund (FWF projects P-28892 and P-33059 to BK and P-34626 to SH and DL).

## Conflicts of interest

There are no conflicts to declare.

## Supplementary Material

CC-061-D5CC02129D-s001

CC-061-D5CC02129D-s002

## Data Availability

See the ESI.‡ Crystallographic data for cupribyrate (Cuby) have been deposited at the Cambridge Crystallographic Data Center (CCDC) and are available under accession number CCDC-2402239.
