# Supplementary material for: Encasing the paramagnetic copper(ii)-ion by the ring-contracted corrin ligand of vitamin B12
Source: Chem Commun (Camb). 2025 Jun 9;61(57):10606–9. doi: 10.1039/d5cc02129d (PMC12174906; doi:10.1039/d5cc02129d)
Supplement: CC-061-D5CC02129D-s001 [file CC-061-D5CC02129D-s001.pdf]

Supplementary Information (SI) for ChemComm.
This journal is © The Royal Society of Chemistry 2025

Encasing the Paramagnetic Copper(II)-Ion by the Ring-Contracted Corrin Ligand of Vitamin B_{12}

Dedicated to the memory of Albert Eschenmoser on the occasion of his 100th birthday

Supplementary Information

Christoph Kieninger[1,2], Klaus Wurst[3], Daniel Leitner[3], Luis P. Peters[2,3], Dennis F. Dinu[2,3], Markus Wiedemair[1,2], Marc-Kevin Zaretzke[4], Martin Bröring[4], Stephan Hohloch[3] Klaus R. Liedl[2,3] & Bernhard Kräutler[1,2]*

[1] Institute of Organic Chemistry, University of Innsbruck, 6020 Innsbruck (Austria);
[2] Center for Molecular Biosciences (CMBI), University of Innsbruck, 6020 Innsbruck (Austria);
[3] Institute of General, Inorganic & Theoretical Chemistry, University of Innsbruck, 6020 Innsbruck (Austria);
[4] Institute for Inorganic & Analytical Chemistry, TU Braunschweig, 38106 Braunschweig (Germany)

Contents

1.General and Abbreviations S2
2. Spectroscopy S2
3. Chromatography....S3
4. Synthesis of cupribyrate (Cuby) from hydrogenobyric acid (Hby)....S4
5. Kinetic experiments of hydrogenobyric acid (Hby) metal chelation....S5
6. EPR-spectroscopic analysis of paramagnetic cupribyric acid (Cuby)....S8
7. Exploratory experiment - Treatment of Cuby with Zn-dust in aqueous NH_4Cl solution at RT....S9
8. Computational analyses S13
9. X-ray crystal structure of Cuby and data analysis....S16
10. References....S43

1. General and Abbreviations:

Abbreviations: RV: rotatory evaporator; RT: room temperature (23 °C); HV: ~$2x10^{-4}$ mbar; NaP7-buffer**:** 10 mM sodium phosphate buffer pH7.

Solvents: acetonitrile (ACN; Fisher, ≥99.8%, HPLC grade); acetic acid (AcOH, Sigma-Aldrich, glacial, ACS reagent, ≥99.7%, distilled under Ar); water (H_2O, Millipore MiliQ); methanol (MeOH; Fisher, ≥99.8%, HPLC grade); acetone (Fluka, puriss. p.a.).

Chemicals: Hydrogenobyric acid (**Hby**);[1] copper acetate monohydrate (Merck, p.a.); activated Zn-dust: Zn-dust (Lab grade), activated by 10 min dispersion in 1M HCl, the re-precipitated Zn was separated off, washed three times with each, H_2O and MeOH, dried under HV and stored under Ar; ammonium chloride ((Sigma Aldrich, ACS reagent ≥99.5%); disodium hydrogenphosphate (Sigma Aldrich, ACS reagent ≥98%); sodium dihydrogenphosphate (Sigma Aldrich, ACS reagent ≥99.5%); argon (Ar 5.0, Messer, ≥99.999%).

Desalting: the (largely aqueous) solution of an isolate was absorbed on a Sep-Pak cartridge, washed with 10 ml of water and the coloured adsorbate was eluted, all, with MeOH.

2. Spectroscopy:

UV-Vis spectra: Agilent Technologies Cary 60; λ_{max} in nm (logε or relative ε), sh signifies shoulder;

CD spectra: Jasco J-1500 CD spectrometer; λ_{max}, λ_{min} and λ_0 in [nm], Δε in [$l \cdot mol^{-1} \cdot cm^{-1}$];

ESI-MS spectra: Finigan LCQ classic and Thermo Scientific LTQ-Orbitrap XL, (+)-ion mode, 4.5 kV; samples were desalted and injected with MeOH; data reported as m/z (intensity in % in relative to base peak, assignment);

EPR spectra: Magnettech ESR5000 X-band benchtop EPR spectrometer equipped with a temperature control unit (microwave-frequency: 9.4712 GHz); measurements performed with 0.5 mg **Cuby** (1.34 mM) in 0.40 ml 20% glycerol in H_2O in a 3 mm o.d. fused silica tube. Simulation of experimental spectrum were done with the EasySpin package for Matlab® and values obtained by last square fitting using the pepper function (see main part, Figure 2 and Fig. S4, for experimental spectra and simulations).

Table S1. Simulation parameters from least square fit of the experimental EPR-Spectrum of **Cuby** at – 125 °C (see main part, Fig. 2)

gx = 2.0378, gy = 2.0376, gz = 2.1404

A(Cu) = 87.3, 80.0, 633 MHz
A(N1) = 43.6, 48.0, 50.8 MHz
A(N2) = 42.2, 43.8, 47.0 MHz
A(N3) = 41.5, 46.3, 47.4 MHz
A(N4) = 46.5, 47.3, 51.4 MHz

3. **Chromatography**:

RP18 cartridges (Waters, Sep-Pak C18 Plus Long, 820 mg); standard conditioning: 10 ml H_2O, 10 ml MeOH, 10 ml ddH_2O. *RP18 column* chromatography. Spherical C18 bonded flash silica (Supelco, 45-75 µm). *HPLC*: Hitachi Elite LaChrom Pump, type L-2130; diode array detector L2450; manual loading of the injection loop (20-100 µl); stationary phase: Phenomenex HyperClone ODS (C18); column parameters: 250 x 4.60 (5 µm; # 755219-4); mobile phase: NaP7-buffer; solvent composition: NaP7-buffer/MeOH: 0-34.5 min (20%-95% MeOH), 34.5-38.5 min (95% MeOH), 38.5-39.5 min (95%-20% MeOH), 39.5 min-44.5min (20% MeOH); flow= 1 mL/min.

4. Synthesis of cupribyrate (Cuby) from hydrogenobyric acid (Hby)

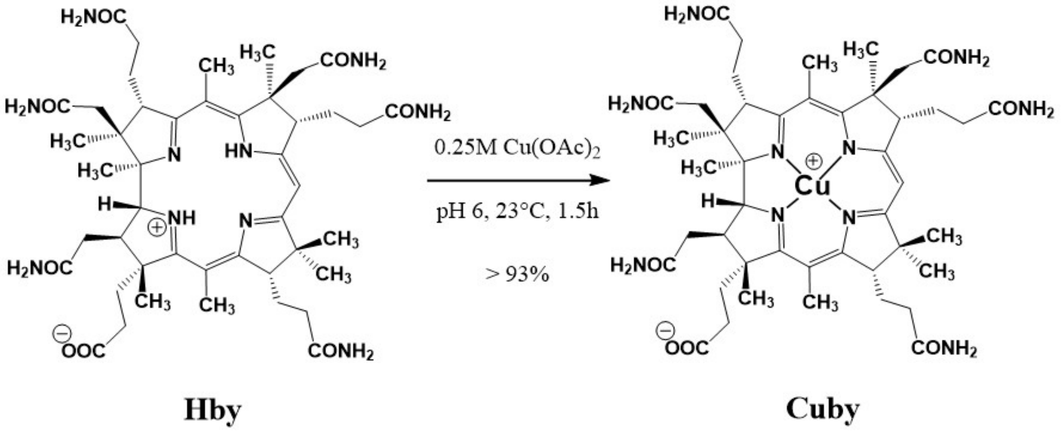

In a 25 ml round bottom flask 3.0 mg (3.43 µmol) crystallized **Hby** [1] was dissolved in 3.45 ml 250mM aqueous $Cu(OAc)_2$ pH 6, resulting in a dark red-brown reaction solution, which was stirred at 23±2°C for 1.5h. The now dark green solution was diluted to about 20ml with H_2O and the mixture was loaded on a conditioned 360mg SepPak cartridge. The adsorbate was washed with 20ml H_2O, 20ml 100mM $NaBF_4$ pH 6 and 20ml H_2O. The **Cuby** was eluted with 3ml 0.1 mM $NaBF_4$ in MeOH and the eluate was dried with a RV (T<40°C). The yellow-orange residue was dissolved in about 90µl H_2O, 10µl of 100mM aqueous $NaBF_4$ pH 6 and 200µl MeCN. The **Cuby** crystallized at RT upon addition of 50µl MeCN and diffusing in MeCN over night at RT, characterized as described below. The resulting semi-crystalline **Cuby** was dissolved in 100µl H_2O and crystallized by the portionwise addition of about 3ml MeCN. The crystals were separated from the ML, and dried overnight using HV. 2.98mg (3.18µmol, 93%) of crystalline **Cuby** was obtained, from which a single crystal was selected for X-ray analysis.

UV/Vis (c=0.014 mMol in NaP7-buffer): λ_{max} [nm] (lg ε)= 484 (4.04), 467 (4.06), 387 (3.68), 367 (3.70), 331 (4.60), 319 (4.32), 291 (4.03), 272 (4.33); see main part, Figure 1.

CD (c=0.019 mM in NaP7-buffer): $\lambda_{max/min}$ [nm] (Δε) = 491 (-7), 331 (55), 318 (21), 291 (-17), 283 (-13), 272 (-30), 233 (9); λ_o [nm]= 399-428, 306, 250; see main part, Figure 1.

HR-ESI-MS (Orbitrap, MeOH, ≥5%): m/z(%) = 940.447 (10), 939.445 (50), 938.444 (78), 937.446 (96), 936.442 (100, $[C_{45}H_{65}CuN_{10}O_8]^+ \hat{=}$ $[M+H]^+$); 496.685 (5), 496.187 (9), 495.684 (9, $[M+H+Na+O]^{2+}$); 481.217 (7); (17), 480.716 (9), 480.218 (16), 479.716 (17, $[M+H+Na]^{2+}$); see Figure S1 (below).

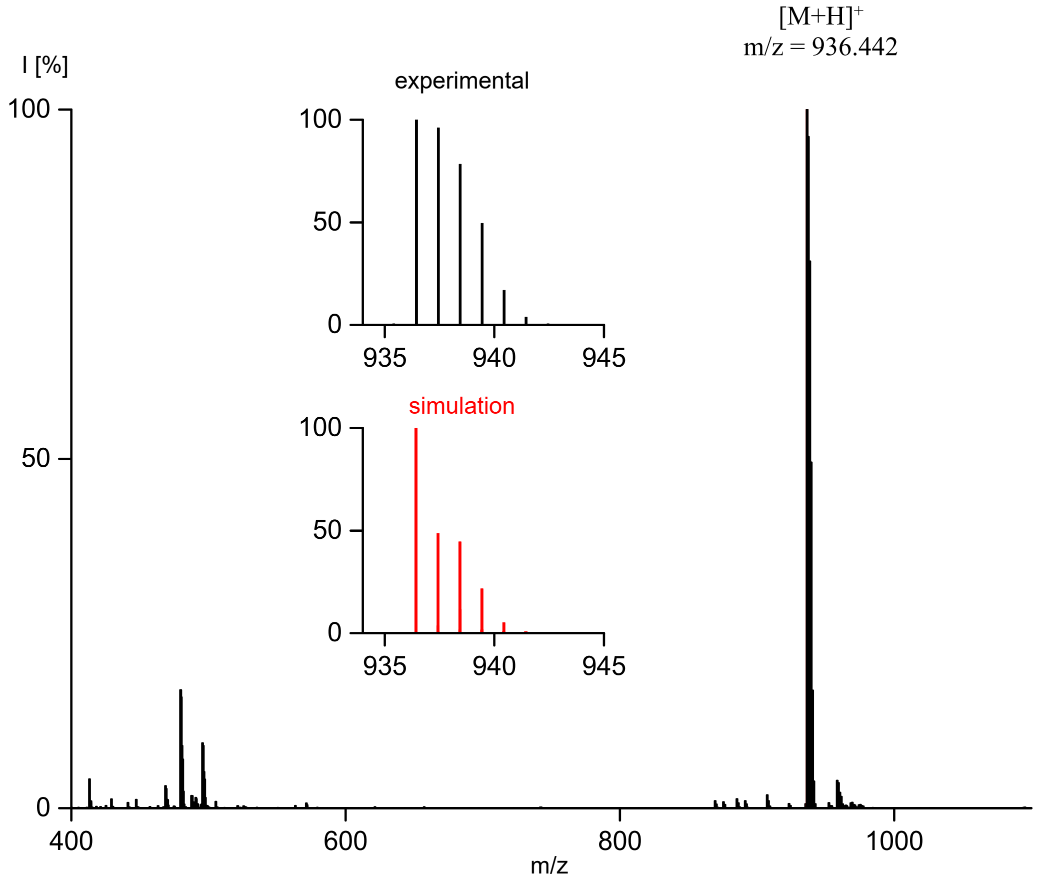

Figure S1: HR-ESI Mass spectrum of cupribyric acid (**Cuby**) in MeOH, 4.5kV

5. Kinetic experiments of hydrogenobyric acid (Hby) chelation with Cu(II)-, Zn(II)- & Co(II)-ions

Kinetic experiments for Cu(II)- and Zn(II)-insertions

Individual samples were prepared from a fresh stock solution of **Hby** (c = 4.19 mM) in water for each analytical time point (time of reaction = time from sample preparation till sampling by injection for analysis by HPLC); experiments were done with air-saturated solutions at RT (21-24°C) and pH 5 containing 0.42 mM **Hby** and 0.10 M $Cu(OAc)_2$ or 0.10 M $Zn(OAc)_2$ for insertion of Cu(II)- or Zn(II)-ions, respectively.

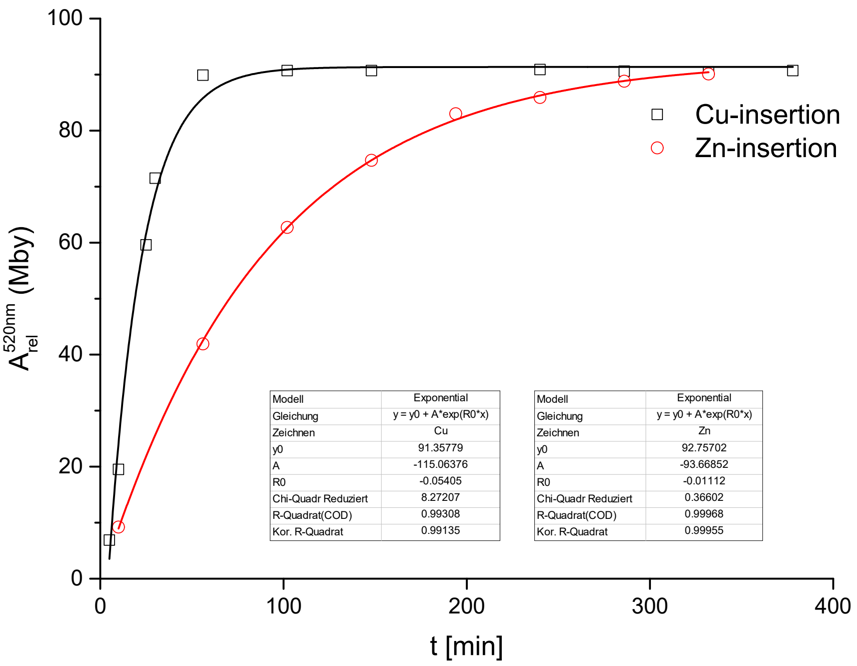

Figure S2. Kinetics of Cu(II)- or Zn(II)-insertions into **Hby** at RT and pH 5 from analysis of UV/Vis-spectroscopic absorbance changes at 520 nm

Table S2. Kinetics and pseudo-first order rates for Cu(II)- and Zn(II)-chelation by **Hby** in aerated aqueous solution (0.1 M $Cu(OAc)_2$, RT, pH 5)

	Cu-insertion	Zn-insertion
$A_{rel}^{520nm}(Mby,\infty)$	91 ± 1	92.8± 0.5
C	-115± 5	-93.7 ± 0.8
**k [min^{-1}]**	**0.054 ± 0.004**	**0.0111 ± 0.0002**
k [s^{-1}]	9.0±0.7*10^{-4}	1.85±0.03*10^{-4}
R^2corr	0.991	1.000

Qualitative analysis of chelation of Co(II)-ions by Hby.

Specific conditions for Co(II)-insertion: 0.42mM **Hby**, 100mM $Co(OAc)_2$ pH 5, aerated solutions at RT; **Hby** underwent partial isomerization and metal-incorporation was not analytically clean; after 3 or 5 days, the Co(II)cobyrate formed was trapped by addition of 100µl 0.1M KCN to the UV/Vis-cell to furnish the stable $(CN)_2$cob(III)yrate (**$(CN)_2$-Cby**), analysed by HPLC- and UV/Vis-spectroscopy (see Fig. S3).

For the Co(II)-chelation by Hby at RT (0.1 M $Co(OAc)_2$ pH5) **a rate k(Co) about 3*10^{-4} min^{-1}** or **3*10^{-3} L.mol^{-1}.min^{-1}** (from pseudo-first order analysis) was estimated.

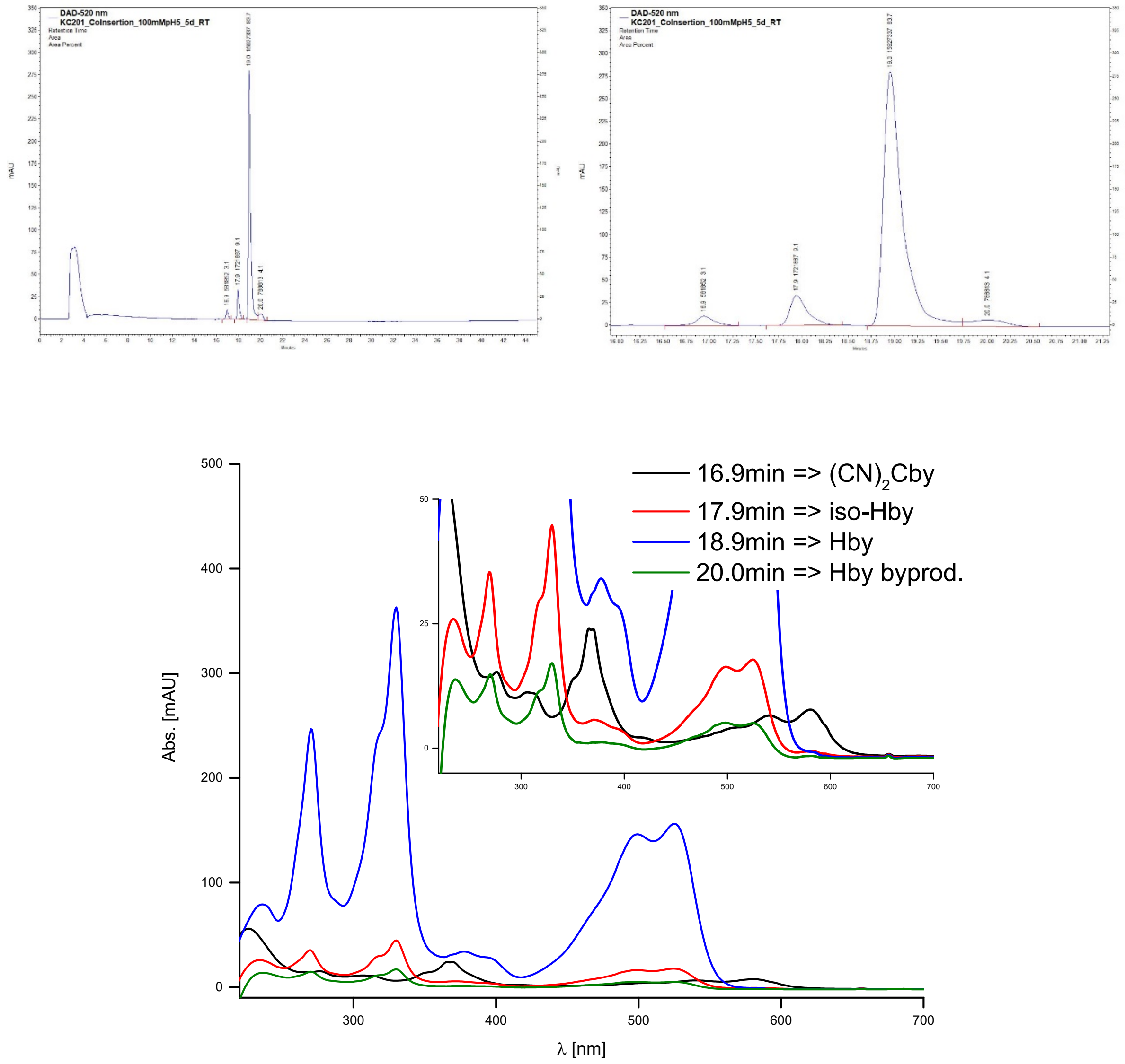

Figure S3. HPLC-Analysis of Co(II)-chelation by **Hby**. Top. HPLC-analysis of reaction mixture after 5 days at RT (left: full scan, right: close-up, detection at 520 nm). A conversion of roughly of 4% is indicated, and a range of side products are detected, all tentatively characterized by on-line UV/Vis-spectra (bottom) of fractions observed at the retention times of 16.9 min (black, **$(CN)_2$-Cby**), 17.9 min (red, ***iso*-Hby**), 18.9 min (blue, **Hby**) and 20.0 min (gray, an unknown **Hby**-analogue); adventitious side products from reactions of the isomerization-labile **Hby** during the 5 day experiment at RT.

Comparison of chelation of Hby with Cu(II)-ions vs. data for Zn(II)- and Co(II)-ions

Cu(II)-ions were chelated by **Hby** in aerated aqueous solutions at RT and at pH5 with a pseudo-first-order rate $k_{obs} = 5.4 \times 10^{-2}$ min^{-1}, corresponding to a rate constant $k^{Cu(II)} = 0.54 \pm 0.04$ $L.mol^{-1}min^{-1}$. Hence, Cu(II)-insertion occurs with a roughly 180 times higher rate than with Co(II)-ions and about 5 times faster than with Zn(II)-ions.

6. EPR-spectroscopic analysis of paramagnetic cupribyric acid (Cuby)

Sample preparation: 1.34 mM solution of **Cuby** in 20% glycerol in H_2O (0.5 mg of **Cuby** in 0.4 ml of the solvent mixture). The coloured clear solution was filled into the 3 mm o.d. fused silica tube and the sample immersed into the cavity of the EPR-spectrometer.
The sample was cooled to -175 °C and measurements were performed at temperature steps of 25 °C. Using optimized parameters the best resolved signal was observed at -125°C.
Measurement parameters: microwave power 20 mW, modulation 0.3 mT, measurement time 240s).

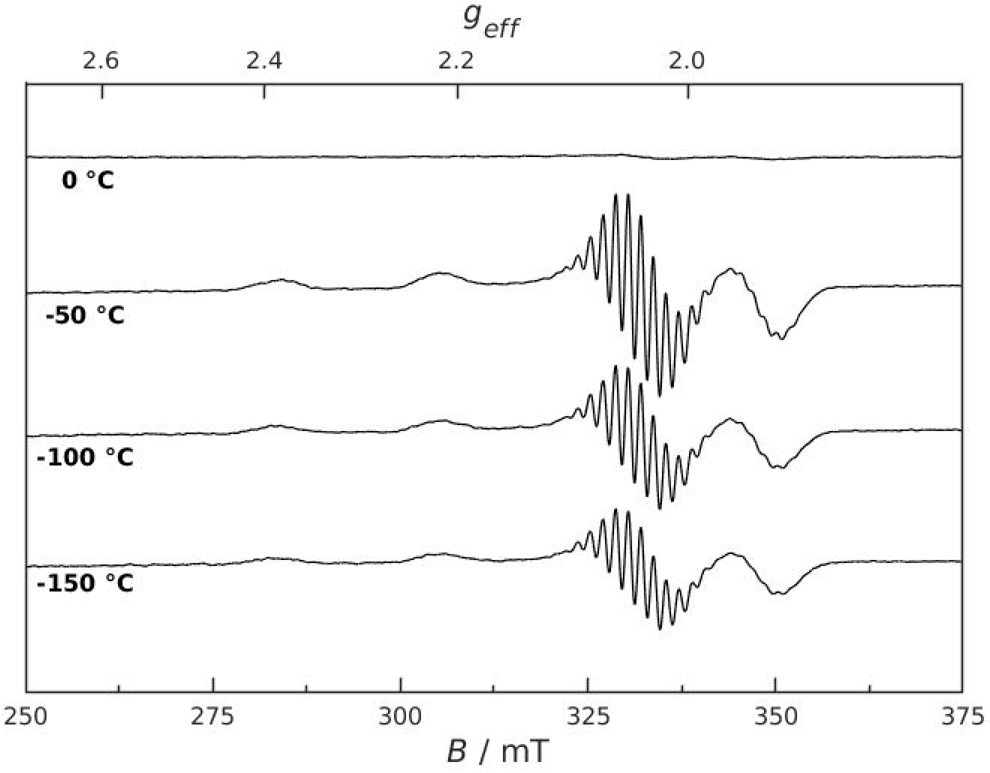

Figure S4. EPR-spectra of a 1.34 mM solution of **Cuby** in H_2O : glycerol (4 :1) cooled to 0 °C, to -50 °C, -100 °C and -150 °C, starting at the lowest temperature. At 0 °C the sample was not frozen and a signal was not visible any longer.

7. Exploratory experiment - Treatment of Cuby with Zn-dust in 1M aqueous NH_4Cl solution at RT

This experiment indicated loss of copper from **Cuby** upon Zn-reduction and formation of the Zn-corrin **Znby** and of provisionally characterized Zn-dihydrocorrin **H_2-Znby** (Scheme S1).

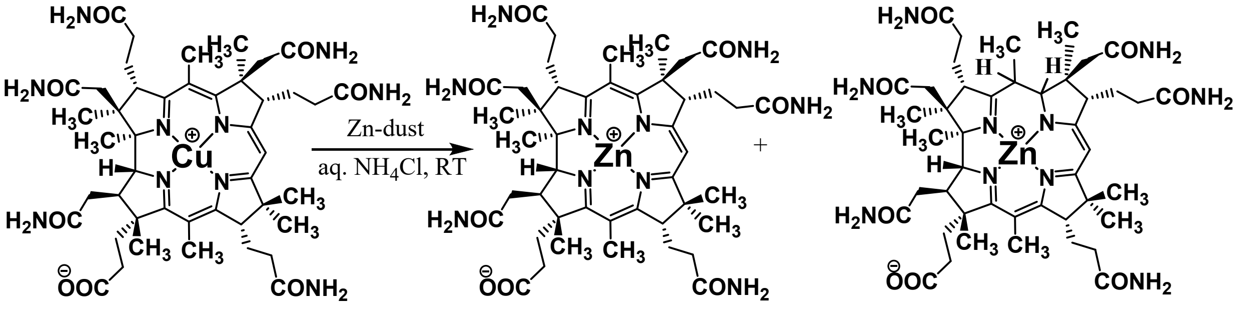

Cuby **Znby** **5,6-H_2-Znby**

Scheme S1. Structural outline of the reduction of **Cuby** by Zn-dust, furnishing the Zn/II)-analogue **Znby** and some of its tentative dihydroform(s), represented here by the proposed structure of a Zn-5,6-dihydrocorrin (**H_2Znby**) with unknown configuration at the now saturated positions 5 and 6.

In a small all glass-combination of a 1mm UV/Vis-cell sealed to a 25 ml round bottom flask containing 10 ml of aqueous 1 M NH_4Cl, 2.0 mg of HPLC-pure **Cuby** were dissolved (pH measured as 5.7) and the solution was saturated with Ar. Then 700 mg of freshly activated Zn dust was added under Ar to the solution, the flask briefly flushed with Ar and UV/Vis-spectra were measured after a reaction time of 15 min, 30 min, 1 h, 2 h, 3 h, 3.5 h and 4 h (see Figure S5). Then the reaction mixture was loaded onto a Sep-Pak cartridge, the adsorbate was washed with water (50 ml) and eluted with 4 : 1 MeOH/water (25 ml). The eluate was concentrated to about 1.5 ml on an RV (< 40 °C) and then loaded onto an RP18 column (1x10 cm, packed in MeOH, conditioned with water, 2 column-volumes each time). The adsorbate was first washed with 20 ml of MeOH/water (1 : 3) and then eluted with 30 ml of MeOH/water (3 : 7). The collected eluate was evaporated to dryness, the residue dissolved in 1 ml of water and analysed via HPLC (Figure S6). The two main fractions, a yellow (greenish-appearing) fraction F1 eluting at 16.4 min (see Figures S7 and S8) and a red-orange fraction F2 at 16.7 min (see Figures S10 and S11), were collected separately, desalted by filtration through a Sep-Pak cartridge and HPLC re-analyzed with on-line UV/Vis-spectra; for F1: see Figures S7 and S8; for F2: see Figures S10 and S11. Both fractions were also analysed via ESI-MS-spectra, which indicated the presence of the main component in F1 compatible with a dihydrozincobyric acid (Figure

S9) and mostly zincobyric acid (**Znby**) and di-oxygenation products of **Znby** (Figure S12) in fraction F2, the latter deduced to be result of adventitious photo-oxygenation during work-up.

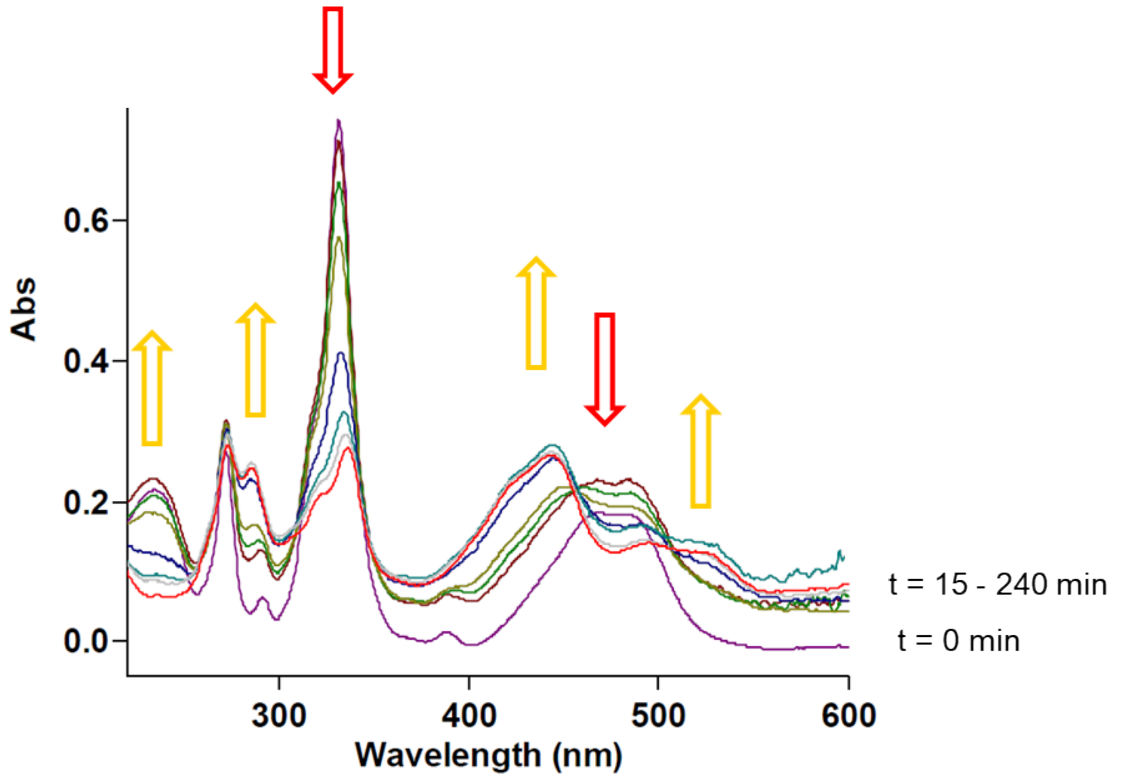

Figure S5. UV/Vis-spectroscopic analysis of the reaction mixture of **Cuby** with Zn-dust in aqueous 1 M NH_4Cl at RT (below named the 'reduction reaction of **Cuby**'). Major absorbance decreases around 480 and 330 nm indicate disappearance of **Cuby**, increases at 290 nm, at 440 nm, and near 520 nm (of **Znby**), indicate formation of **H_2-Znby** and **Znby**.

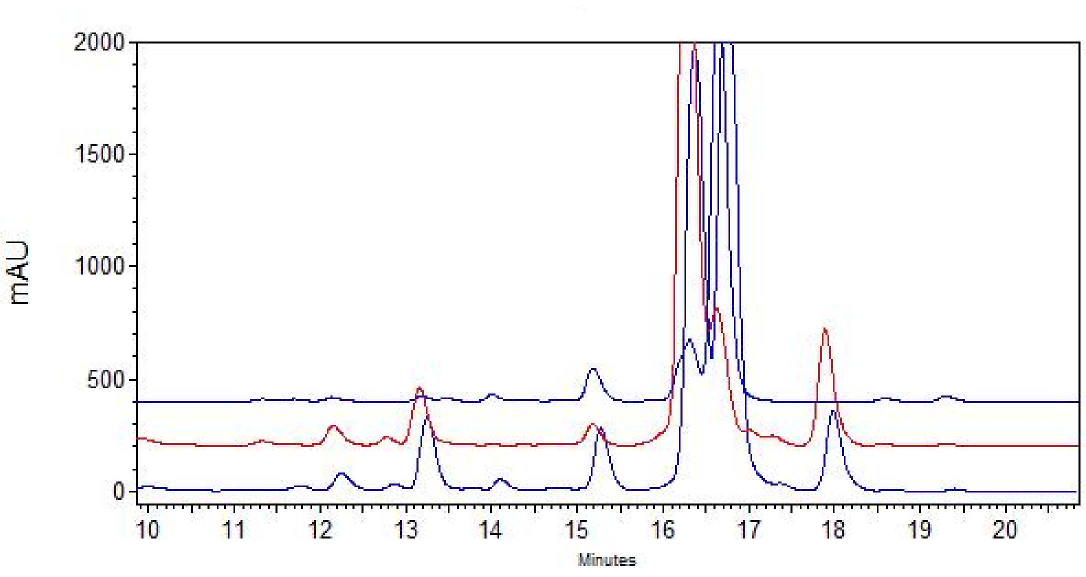

Figure S6. HPLC-analysis of the reduction reaction of Cuby with detection, top to bottom, at 490 (blue line), 440 (red line) and 270 nm (blue line). The two major fractions at retention times of 16.4 and 16.7 min were identified as a dihydro-Zn(II)-corrin and the Zn(II)-corrin

Znby, respectively (see below); residual **Cuby**, as minor fraction at a 15.2 min retention time, indicated by on-line UV/vis.

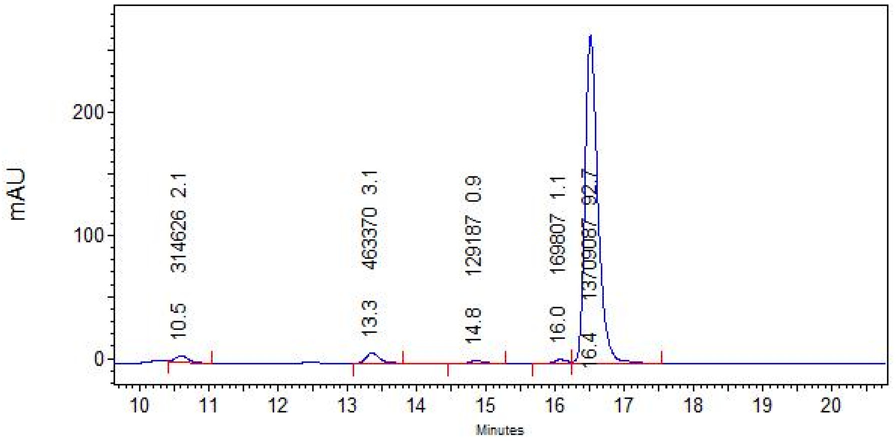

Figure S7. HPLC-re-analysis of the more polar HPLC-fraction F1 (collected at 16.4 min) of the reduction reaction mixture of **Cuby** (detection at 440 nm).

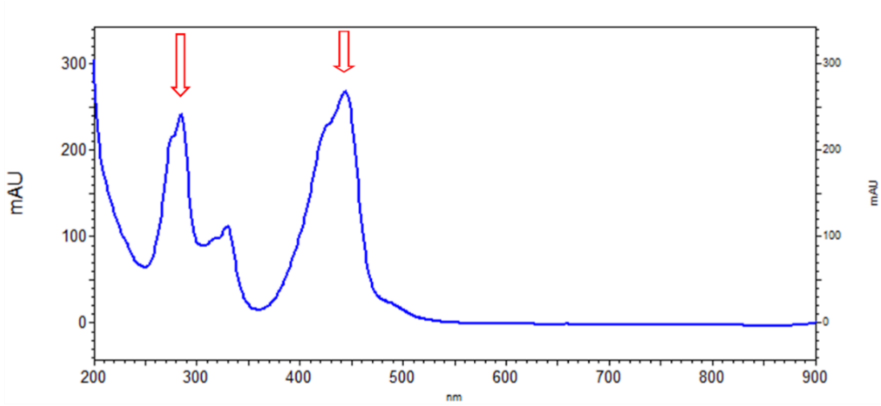

Figure S8. UV/Vis-spectrum of the HPLC-collected fraction F1 of the reduction reaction mixture of **Cuby**. The highlighted absorption maxima at 440 and 287 nm (shoulder at 425 nm) and with rel. intensities of 1.0 : 0.87 : 0.87, indicate a type of 'yellow corrinoid' as its main component. [2, 3]

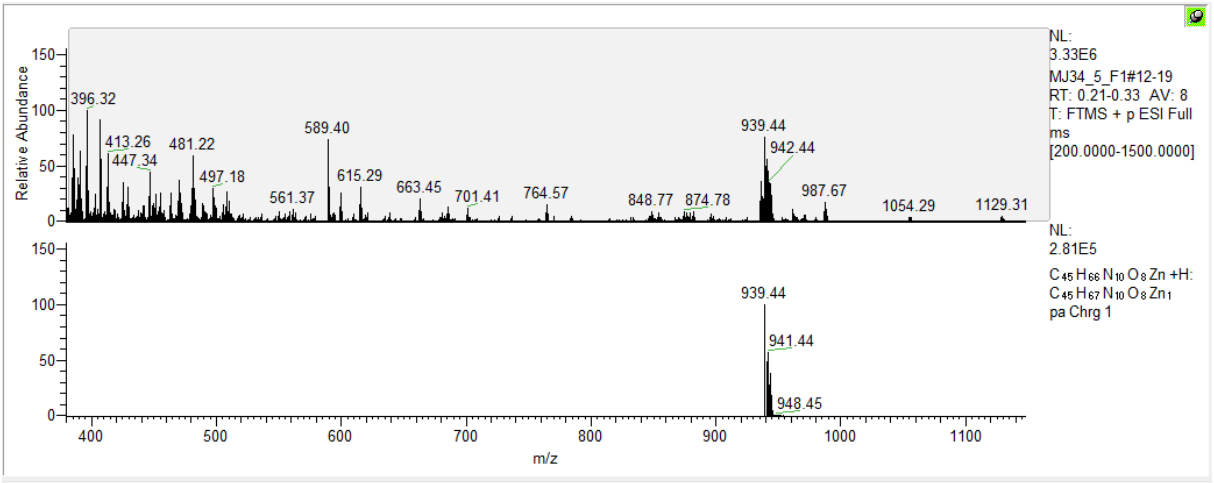

Figure S9. Mass-spectrometric analysis of the HPLC-collected fraction F1 from the reduction reaction mixture of **Cuby**. The detected main signals are compatible with the molecular

formula $C_{45}H_{66}N_{10}O_8Zn$ of a dihydro-Zn-corrin (**H_2Znby**); in this sample, **Cuby** (see Fig. S1) was not indicated, nor major amounts of a dihydro-Cu-corrin.

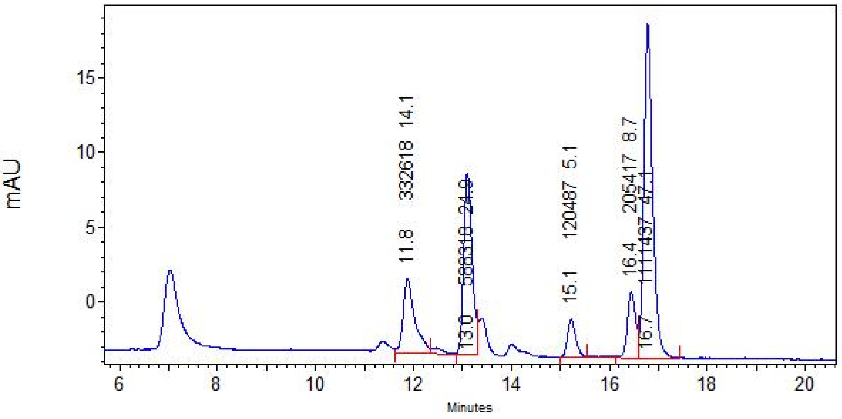

Figure S10. HPLC-re-analysis of the less polar HPLC-fraction F2 of the reduction reaction mixture of **Cuby**, collected at around 16.7 min (detection at 440 nm). The major fraction at a retention time of 16.7 min corresponds to **Znby** (see Figs. S11 and S12), the minor fraction at retention time of 15.1 min is compatible with residual **Cuby**.

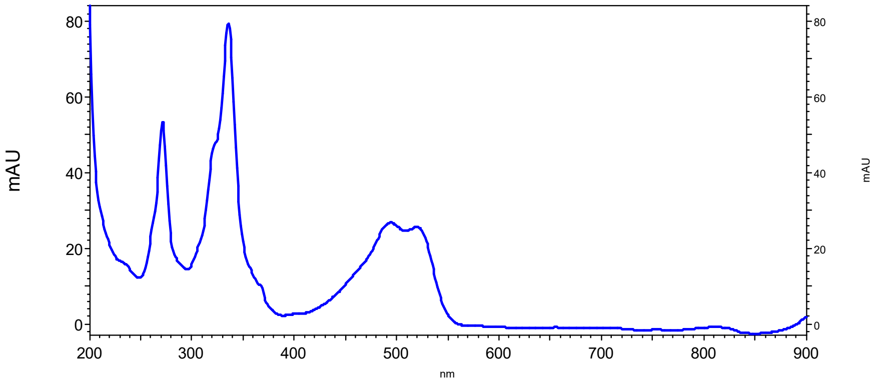

Figure S11. On-line UV/Vis-spectrum at 16.7 min of the HPLC-re-analysis of the isolated less polar fraction F2 of the reduction reaction mixture of **Cuby** (see Figure S10), featuring the absorption characteristics of **Znby** [4].

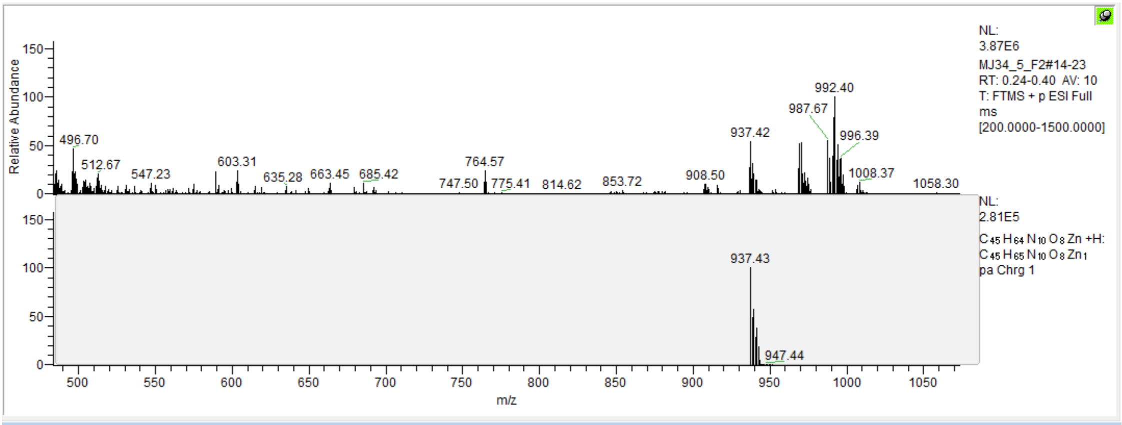

Figure S12. Mass-spectrometric analysis of the HPLC-collected fraction F2 of the reduction reaction mixture of **Cuby**. The observed signals signify the absence of bound copper in the here analyzed fraction. The ion at m/z = 937.42 indicates the molecular formula

$C_{45}H_{64}N_{10}O_8Zn$, due to the Zn-corrin **Znby**; ions around m/z = 969 (+ O_2), at 987.67 (+ O_2 + H_2O) and around 991 (+ 2 O_2) indicated adventitious photoaddition of dioxygen to **Znby** in the isolate by efficient Znby-photosensitized formation of singlet oxygen during work-up.

8. Computational analyses

Methodology

From the X-ray crystallographic data of cupribyrate (**Cuby**), cartesian atomic coordinates (xyz) were used as the best experimental basis for an equilibrium structure, and water and solvent molecules were removed. The restricted closed-shell and open-shell Hartree-Fock Self-Consistent Field (SCF) with the Resolution of Identity approximation calculations were computed with Molpro [5] using Dunning all-electron basis set cc-pVDZ [6] and, for copper, the aug-cc-pVDZ basis set [7, 8]. Transition metals often exhibit mixed spin states. Thus, from a theoretical point of view, multi-configuration or complete active space SCF methods would be advisable. However, those would not change the qualitative interpretation of frontier molecular orbitals as done here. The Hartree-Fock SCF MOs were visualized in the IBOView program [9] and identified visually as π systems.

A range of the in gas-phase calculated MOs of cupribyric acid (**HCuby**$^+$) in comparison with cupribyrate (**Cuby**) are shown below, in Figure ST1; three frontier molecular orbitals (MO) of **HCuby**$^+$ are also depicted in Figure 4 (main text). In the here studied carboxylate-protonated form **HCuby**$^+$ of **Cuby** artefactual contributions to the mostly π-based calculated corrin MO's are absent, which typically occur in gas-phase calculations.

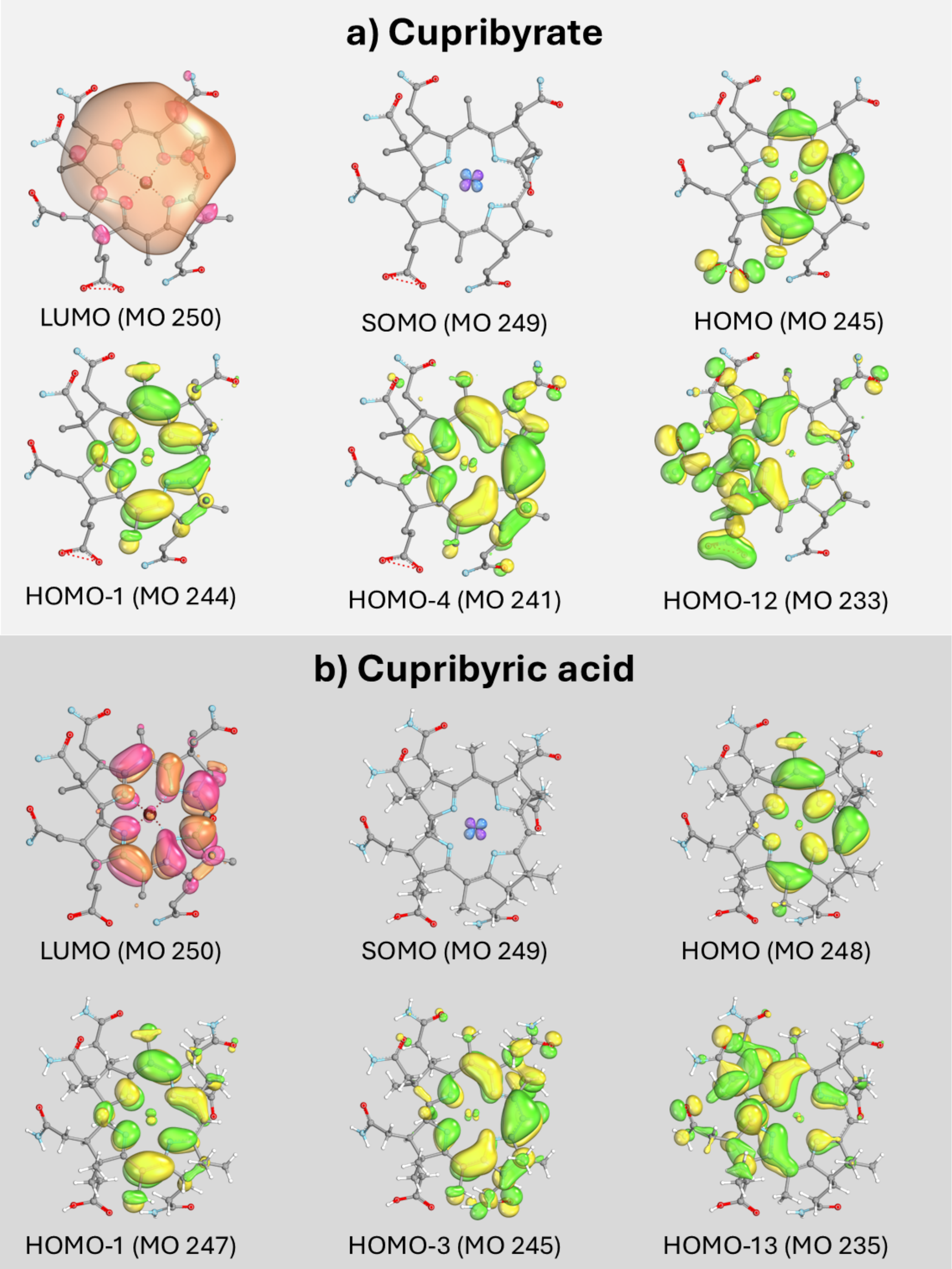

Figure S13: Molecular orbitals of a) cupribyrate (**Cuby**) and b) the cupribyric acid cation (**HCuby**$^{+}$) from SCF calculations based on the atom coordinates of the crystal structure (the view is from the upper side).

In view of the here disclosed reduction-induced removal of the copper-center of **Cuby**, the experimentally still elusive cuprobyrate anion (**Cu(I)by**$^{-}$) was tested in models by placing a 4-coordinate Cu(I)-ion into the known structures of nibyric acid (**Niby**),[10] **Cuby**, and zincobyric acid (**Znby**) [4]. The Cu(I)-in-**Cuby** is energetically lower than the Cu(I)-in-**Znby**, while Cu(I)-in-**Niby** does not converge, indicative of a highly strained geometry imposed by the exceptionally small coordination hole provided by the corrin ring in **Niby**. We further compared our hypothetical Cu(I)-in-**Cuby** with **Cuby** in a scan of the potential energy curve (PEC) along the perpendicular (axial) movement of the copper ion vs. the corrin ligand (see Figure S14). As expected, the energy increases significantly in both directions for **Cuby**. For Cu(I)-in-**Cuby**, the energy initially decreases in both directions, passing through minima near 0.8 Å above (lower minimum) or 0.8 Å below the best corrin plane, before increasing again with further dislocation. This suggests that the Cu(I)-in-**Cuby** geometry is instable and a better hypothetical **Cu(I)by** structure would be to place the Cu(I)-ion slightly further away from the corrin ring, similar to the experimental **Znby** structure.

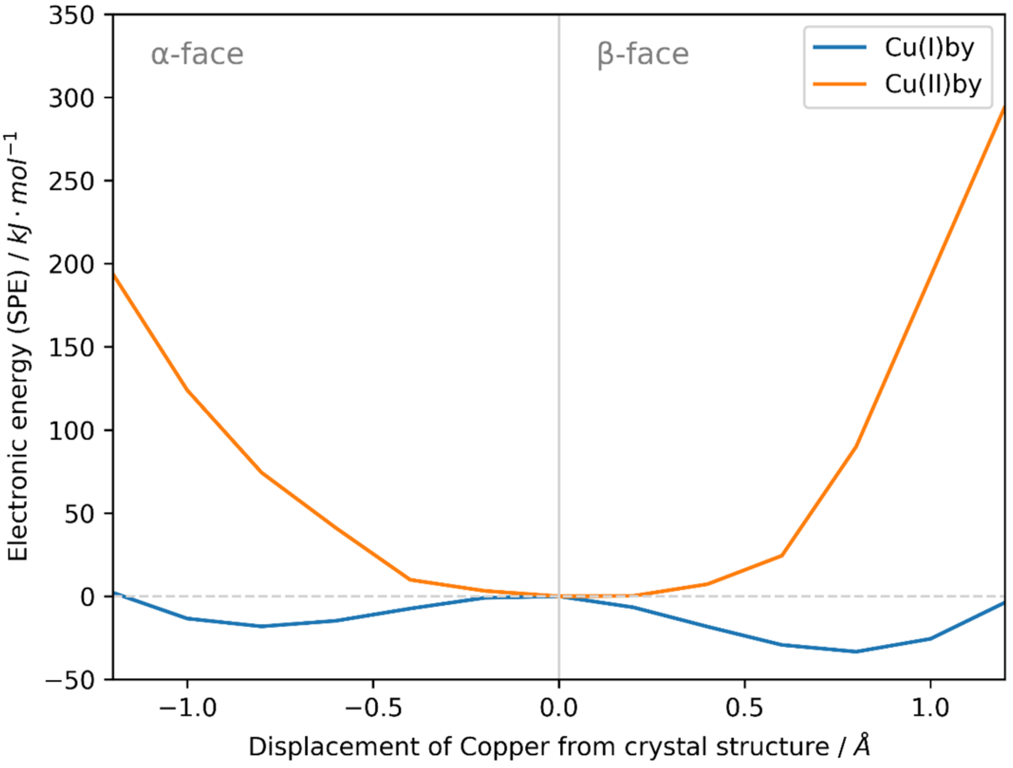

Figure S14. Calculated potential energy surfaces for the Cu(II)-corrin cupribyrate (**Cuby**) and the Cu(I)-analogue cuprobyrate (**Cu(I)by**) with axial displacement of the copper center from the best plane through the four inner corrin nitrogens, based on the crystal structure of cupribyrate (**Cuby**).

9. X-ray crystal structure of Cuby and data analysis

A single crystal of **Cuby**, grown, as described above, from H_2O/MeCN at ambient temperature, was used for the crystal structure analysis with data collected at 183K with a Bruker D8 Quest diffractometer (Photon 100 detector) equipped with a microfocus source generator combined with multi-layer optics (monochromatized Mo Kα radiation, λ = 71.073 pm). The structure was solved with SHELXT (version 2014/11) [11] and structure refinement (full-matrix least-squares against F^2) with SHELXL (version 2014/10) [12]. Relevant details of the data collection and data evaluation are listed in Table S3 (see main text, Figures 3 and 4, and Figure S3) Supporting crystallographic data of **Cuby** may be obtained from the Cambridge Crystallographic Data Centre CCDC deposition service via www.ccdc.cam.ac.uk/structures on quoting the deposition number CCDC 2402239.

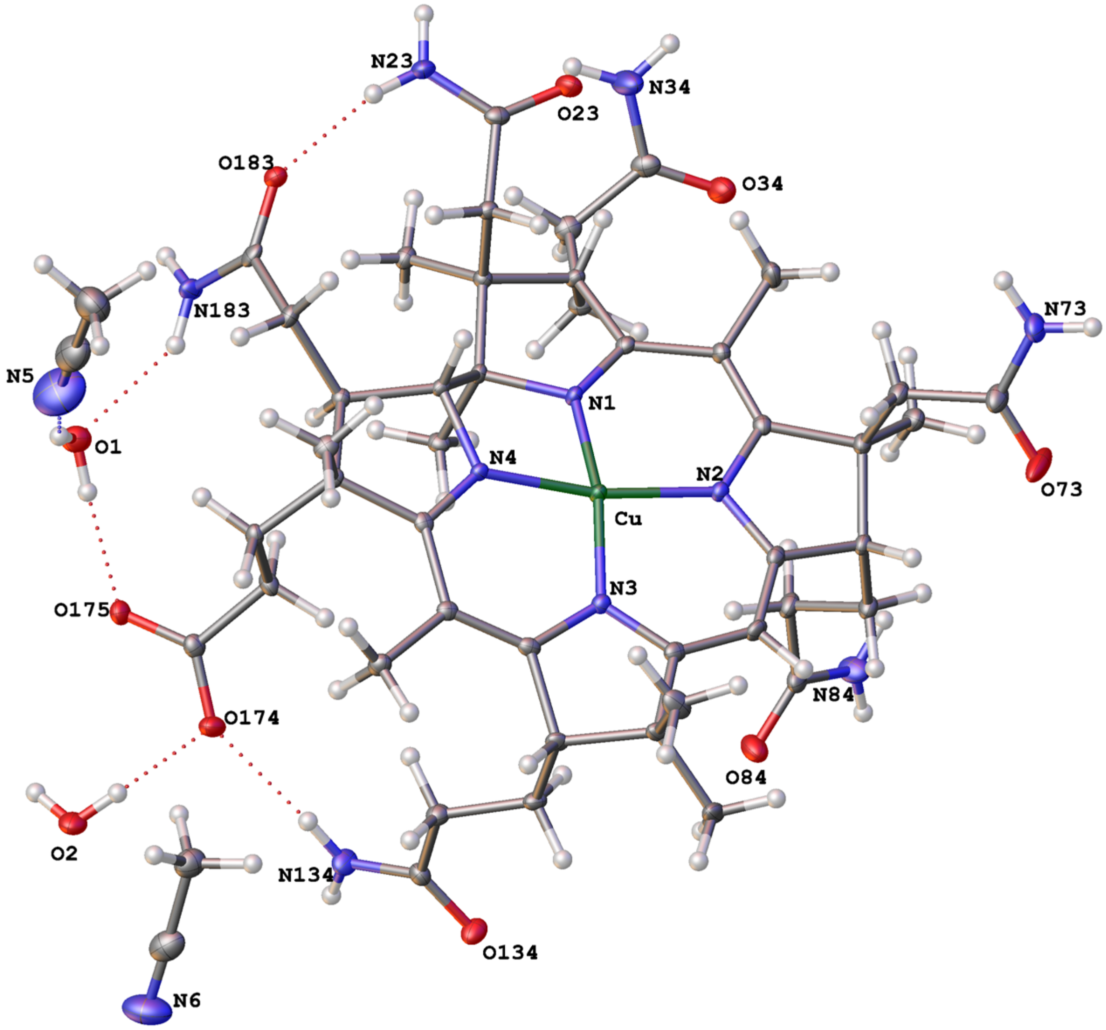

Figure S15. ORTEP-generated model of the crystal structure of **Cuby** and its neighbouring solvent molecules, including ordered H-bonded water and acetonitrile.

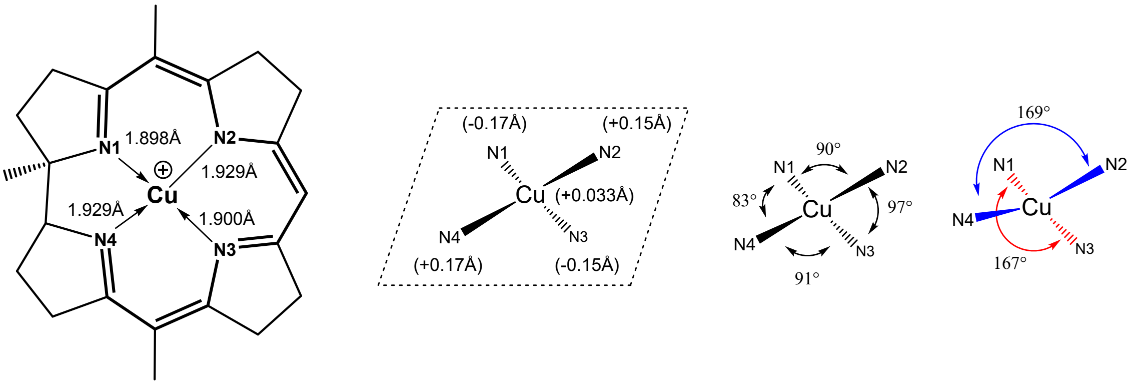

Figure S16. Selected X-ray crystallographic bonding characteristics around the Cu(II)-ion of **Cuby**

Table S3. Comparison of key structural characteristics of **Hby**,[1] **Znby**,[4] **Cuby**, and **Niby**,[10] extracted from their X-ray crystal structures (for more details see the cited references).

	**Hby**	**Znby**	**Cuby**	**Niby**
Corrin-fold [13]	11.6°	6.38°	10.0°	8.7°
Corrin-helicity [1]	12.9°	7.97°	12.4°	10.1°
Interplanar angle at metal-ion [1]	13.5°	50.2°	13.6°	11.0°
Bond length **M-N1**		2.039 Å	1.898 Å	1.836 Å
Bond length **M-N2**		2.031 Å	1.929 Å	1.882 Å
Bond length **M-N3**		1.986 Å	1.900 Å	1.864 Å
Bond length **M-N4**		2.054 Å	1.929 Å	1.859 Å
Distance **N1-4N-plane**	-0.17 Å	-0.107 Å	- 0.17 Å	- 0.13 Å
Distance **N2-4N-plane**	+0.15 Å	+0.096 Å	+ 0.15 Å	+ 0.12 Å
Distance **N3-4N-plane**	-0.14 Å	-0.094 Å	- 0.15 Å	- 0.12 Å
Distance **N4-4N-plane**	+0.17 Å	+0.104 Å	+ 0.17 Å	+ 0.13 Å
Distance **M-4N-plane**		+0.624 Å	+ 0.033 Å	+ 0.025 Å
Diagonal distance **N1-N3**	3.668 Å	3.751 Å	3.773 Å	3.683 Å
Diagonal distance **N2-N4**	3.965 Å	3.937 Å	3.841 Å	3.730 Å

Table S4. Crystal data and structure refinement for Cupribyric acid (Cuby) crystallized from H_2O and acetonitrile at ambient temperature.

Empirical formula	$C_{45}H_{64}CuN_{10}O_8$ x 2 H_2O x 2 CH_3CN	
Formula weight	1054.74	
Temperature	183(2) K	
Wavelength	0.71073 Å	
Crystal system	Monoclinic	
Space group	$P2_1$ (no. 4)	
Unit cell dimensions	a = 7.0448(3) Å	α= 90°.
	b = 24.6484(12) Å	β= 94.9200(10)°.
	c = 15.2708(7) Å	γ = 90°.
Volume	2641.9(2) $Å^3$	
Z	2	
Density (calculated)	1.326 Mg/m^3	
Absorption coefficient	0.481 mm^{-1}	
F(000)	1122	
Crystal size	0.160 x 0.080 x 0.040 mm^3	
Theta range for data collection	2.677 to 24.996°.	
Index ranges	-8<=h<=8, -29<=k<=29, -18<=l<=18	
Reflections collected	33821	
Independent reflections	9307 [R(int) = 0.0264]	
Completeness to theta = 24.996°	99.9 %	
Absorption correction	Semi-empirical from equivalents	
Max. and min. transmission	0.914 and 0.862	
Refinement method	Full-matrix least-squares on F^2	
Data / restraints / parameters	9307 / 5 / 670	
Goodness-of-fit on F^2	1.034	
Final R indices [I>2sigma(I)]	R1 = 0.0272, wR2 = 0.0693	
R indices (all data)	R1 = 0.0298, wR2 = 0.0706	
Absolute structure parameter	-0.010(3)	
Extinction coefficient	0.0075(8)	
Largest diff. peak and hole	0.300 and -0.220 e.$Å^{-3}$	

Table S5. Atomic coordinates (x 10^4) and equivalent isotropic displacement parameters ($Å^2$x 10^3) for **Cuby**. U(eq) is defined as one third of the trace of the orthogonalized U^{ij} tensor.

	x	y	z	U(eq)
Cu	4708(1)	4456(1)	2554(1)	17(1)
N(1)	2949(3)	5023(1)	2251(2)	17(1)
N(2)	4687(3)	4252(1)	1334(2)	17(1)
N(3)	6033(3)	3837(1)	3031(2)	18(1)
N(4)	4804(3)	4796(1)	3695(2)	16(1)
N(23)	1653(4)	7186(1)	2680(2)	29(1)
N(34)	-1931(6)	6632(1)	99(2)	50(1)
N(73)	8083(4)	4781(1)	-1680(2)	35(1)
N(84)	-567(4)	2787(1)	-391(2)	37(1)
N(134)	1318(4)	2601(1)	5496(2)	30(1)
N(183)	716(4)	6258(1)	5420(2)	26(1)
O(23)	2058(4)	6844(1)	1341(2)	32(1)
O(34)	-2024(4)	5826(1)	-569(2)	38(1)
O(73)	7175(5)	3929(1)	-1504(2)	68(1)
O(84)	458(4)	2529(1)	979(2)	42(1)
O(134)	2535(3)	2001(1)	4588(2)	32(1)
O(174)	1501(4)	3646(1)	6487(2)	34(1)
O(175)	650(3)	4458(1)	6962(1)	28(1)
O(183)	2124(3)	6821(1)	4510(2)	25(1)
C(1)	2124(4)	5295(1)	3004(2)	16(1)
C(1A)	641(4)	4908(1)	3343(2)	21(1)
C(2A)	-512(4)	6028(1)	2998(2)	21(1)
C(2)	1234(4)	5824(1)	2570(2)	16(1)
C(3)	819(4)	5652(1)	1581(2)	17(1)
C(4)	2300(4)	5213(1)	1498(2)	17(1)
C(5)	2891(4)	5016(1)	665(2)	19(1)
C(6)	3913(4)	4548(1)	610(2)	18(1)
C(7)	4537(4)	4281(1)	-231(2)	20(1)
C(7A)	3090(5)	4289(1)	-1042(2)	27(1)
C(8)	5046(4)	3698(1)	110(2)	20(1)
C(9)	5487(4)	3791(1)	1078(2)	19(1)
C(10)	6483(4)	3422(1)	1630(2)	22(1)
C(11)	6721(4)	3443(1)	2537(2)	19(1)
C(12B)	9975(5)	3234(2)	3189(2)	34(1)

C(12)	7890(4)	3037(1)	3109(2)	22(1)
C(12A)	7814(6)	2454(1)	2756(2)	34(1)
C(13)	7016(4)	3127(1)	3995(2)	20(1)
C(14)	6413(4)	3720(1)	3924(2)	18(1)
C(15)	6206(4)	4072(1)	4606(2)	17(1)
C(16)	5349(4)	4609(1)	4471(2)	16(1)
C(17)	5003(4)	5023(1)	5190(2)	17(1)
C(17B)	6928(4)	5282(1)	5500(2)	23(1)
C(18)	3605(4)	5440(1)	4682(2)	16(1)
C(19)	3883(4)	5337(1)	3704(2)	15(1)
C(21)	2793(4)	6272(1)	2592(2)	18(1)
C(22)	2133(4)	6795(1)	2146(2)	22(1)
C(31)	-1167(4)	5421(1)	1273(2)	23(1)
C(32)	-2656(4)	5848(1)	954(2)	27(1)
C(33)	-2189(5)	6100(1)	92(2)	27(1)
C(51)	2373(5)	5374(1)	-130(2)	30(1)
C(71)	6386(5)	4576(1)	-425(2)	26(1)
C(72)	7246(4)	4401(2)	-1253(2)	31(1)
C(81)	3478(4)	3264(1)	-33(2)	23(1)
C(82)	1681(5)	3358(1)	456(2)	27(1)
C(83)	450(5)	2857(1)	375(2)	25(1)
C(131)	5270(4)	2756(1)	4085(2)	21(1)
C(132)	4325(5)	2816(1)	4933(2)	24(1)
C(133)	2643(5)	2438(1)	4995(2)	23(1)
C(151)	6854(5)	3891(1)	5530(2)	24(1)
C(171)	4004(4)	4790(1)	5968(2)	18(1)
C(172)	2313(4)	4421(2)	5676(2)	22(1)
C(173)	1423(4)	4155(1)	6440(2)	22(1)
C(181)	3894(4)	6027(1)	5026(2)	21(1)
C(182)	2158(5)	6401(1)	4957(2)	22(1)
O(1)	692(4)	5569(1)	6952(2)	37(1)
O(2)	-297(4)	3258(1)	7836(2)	38(1)
N(5)	4454(8)	6001(2)	7606(4)	93(2)
C(23)	5836(8)	6249(2)	7596(3)	57(1)
C(24)	7564(7)	6560(2)	7558(3)	59(1)
N(6)	3014(6)	2127(2)	8127(3)	70(1)
C(25)	3762(6)	2496(2)	7883(3)	44(1)
C(26)	4704(5)	2977(2)	7590(3)	39(1)

Table S6. Bond lengths [Å] for crystalline **Cuby**

Cu-N(1)	1.898(2)
Cu-N(3)	1.900(2)
Cu-N(4)	1.929(2)
Cu-N(2)	1.929(2)
N(1)-C(4)	1.289(4)
N(1)-C(1)	1.491(4)
N(2)-C(9)	1.343(4)
N(2)-C(6)	1.395(4)
N(3)-C(11)	1.346(4)
N(3)-C(14)	1.397(4)
N(4)-C(16)	1.298(4)
N(4)-C(19)	1.483(4)
N(23)-C(22)	1.325(4)
N(23)-H(23A)	0.8800
N(23)-H(23B)	0.8800
N(34)-C(33)	1.324(5)
N(34)-H(34A)	0.8800
N(34)-H(34B)	0.8800
N(73)-C(72)	1.310(5)
N(73)-H(73A)	0.8800
N(73)-H(73B)	0.8800
N(84)-C(83)	1.329(4)
N(84)-H(84A)	0.8800
N(84)-H(84B)	0.8800
N(134)-C(133)	1.318(4)
N(134)-H(13A)	0.8800
N(134)-H(13B)	0.8800
N(183)-C(182)	1.334(4)
N(183)-H(18A)	0.8800
N(183)-H(18B)	0.8800
O(23)-C(22)	1.231(4)
O(34)-C(33)	1.229(4)
O(73)-C(72)	1.224(5)
O(84)-C(83)	1.227(4)
O(134)-C(133)	1.243(4)
O(174)-C(173)	1.256(4)
O(175)-C(173)	1.252(4)

O(183)-C(182)	1.239(4)
C(1)-C(1A)	1.539(4)
C(1)-C(2)	1.568(4)
C(1)-C(19)	1.569(4)
C(1A)-H(1A1)	0.9800
C(1A)-H(1A2)	0.9800
C(1A)-H(1A3)	0.9800
C(2A)-C(2)	1.526(4)
C(2A)-H(2A1)	0.9800
C(2A)-H(2A2)	0.9800
C(2A)-H(2A3)	0.9800
C(2)-C(21)	1.557(4)
C(2)-C(3)	1.572(4)
C(3)-C(4)	1.516(4)
C(3)-C(31)	1.546(4)
C(3)-H(3)	1.0000
C(4)-C(5)	1.455(4)
C(5)-C(6)	1.367(4)
C(5)-C(51)	1.520(4)
C(6)-C(7)	1.541(4)
C(7)-C(7A)	1.536(4)
C(7)-C(71)	1.543(4)
C(7)-C(8)	1.559(4)
C(7A)-H(7A1)	0.9800
C(7A)-H(7A2)	0.9800
C(7A)-H(7A3)	0.9800
C(8)-C(9)	1.503(4)
C(8)-C(81)	1.540(4)
C(8)-H(8)	1.0000
C(9)-C(10)	1.389(4)
C(10)-C(11)	1.381(4)
C(10)-H(10)	0.9500
C(11)-C(12)	1.523(4)
C(12B)-C(12)	1.543(5)
C(12B)-H(12A)	0.9800
C(12B)-H(12B)	0.9800
C(12B)-H(12C)	0.9800
C(12)-C(12A)	1.536(4)
C(12)-C(13)	1.550(4)

C(12A)-H(12D)	0.9800
C(12A)-H(12E)	0.9800
C(12A)-H(12F)	0.9800
C(13)-C(14)	1.522(4)
C(13)-C(131)	1.548(4)
C(13)-H(13)	1.0000
C(14)-C(15)	1.374(4)
C(15)-C(16)	1.462(4)
C(15)-C(151)	1.512(4)
C(16)-C(17)	1.534(4)
C(17)-C(17B)	1.537(4)
C(17)-C(171)	1.543(4)
C(17)-C(18)	1.579(4)
C(17B)-H(17A)	0.9800
C(17B)-H(17B)	0.9800
C(17B)-H(17C)	0.9800
C(18)-C(19)	1.543(4)
C(18)-C(181)	1.548(4)
C(18)-H(18)	1.0000
C(19)-H(19)	1.0000
C(21)-C(22)	1.513(4)
C(21)-H(21A)	0.9900
C(21)-H(21B)	0.9900
C(31)-C(32)	1.536(4)
C(31)-H(31A)	0.9900
C(31)-H(31B)	0.9900
C(32)-C(33)	1.517(5)
C(32)-H(32A)	0.9900
C(32)-H(32B)	0.9900
C(51)-H(51A)	0.9800
C(51)-H(51B)	0.9800
C(51)-H(51C)	0.9800
C(71)-C(72)	1.511(4)
C(71)-H(71A)	0.9900
C(71)-H(71B)	0.9900
C(81)-C(82)	1.541(4)
C(81)-H(81A)	0.9900
C(81)-H(81B)	0.9900
C(82)-C(83)	1.507(4)

C(82)-H(82A)	0.9900
C(82)-H(82B)	0.9900
C(131)-C(132)	1.513(4)
C(131)-H(13C)	0.9900
C(131)-H(13D)	0.9900
C(132)-C(133)	1.516(4)
C(132)-H(13E)	0.9900
C(132)-H(13F)	0.9900
C(151)-H(15A)	0.9800
C(151)-H(15B)	0.9800
C(151)-H(15C)	0.9800
C(171)-C(172)	1.534(4)
C(171)-H(17D)	0.9900
C(171)-H(17E)	0.9900
C(172)-C(173)	1.520(4)
C(172)-H(17F)	0.9900
C(172)-H(17G)	0.9900
C(181)-C(182)	1.528(4)
C(181)-H(18C)	0.9900
C(181)-H(18D)	0.9900
O(1)-H(1OA)	0.83(3)
O(1)-H(1OB)	0.84(3)
O(2)-H(2OB)	0.82(3)
O(2)-H(2OA)	0.82(3)
N(5)-C(23)	1.151(7)
C(23)-C(24)	1.444(7)
C(24)-H(24A)	0.9800
C(24)-H(24B)	0.9800
C(24)-H(24C)	0.9800
N(6)-C(25)	1.131(6)
C(25)-C(26)	1.448(6)
C(26)-H(26A)	0.9800
C(26)-H(26B)	0.9800
C(26)-H(26C)	0.9800

Table S7. Bond angles [°] for crystalline **Cuby**

N(1)-Cu-N(3)	166.69(10)
N(1)-Cu-N(4)	82.65(10)
N(3)-Cu-N(4)	91.42(10)
N(1)-Cu-N(2)	90.31(10)
N(3)-Cu-N(2)	97.10(10)
N(4)-Cu-N(2)	169.22(10)
C(4)-N(1)-C(1)	113.1(2)
C(4)-N(1)-Cu	131.3(2)
C(1)-N(1)-Cu	115.65(17)
C(9)-N(2)-C(6)	111.0(2)
C(9)-N(2)-Cu	122.2(2)
C(6)-N(2)-Cu	126.81(19)
C(11)-N(3)-C(14)	110.5(2)
C(11)-N(3)-Cu	123.6(2)
C(14)-N(3)-Cu	125.91(19)
C(16)-N(4)-C(19)	113.9(2)
C(16)-N(4)-Cu	130.93(19)
C(19)-N(4)-Cu	114.66(17)
C(22)-N(23)-H(23A)	120.0
C(22)-N(23)-H(23B)	120.0
H(23A)-N(23)-H(23B)	120.0
C(33)-N(34)-H(34A)	120.0
C(33)-N(34)-H(34B)	120.0
H(34A)-N(34)-H(34B)	120.0
C(72)-N(73)-H(73A)	120.0
C(72)-N(73)-H(73B)	120.0
H(73A)-N(73)-H(73B)	120.0
C(83)-N(84)-H(84A)	120.0
C(83)-N(84)-H(84B)	120.0
H(84A)-N(84)-H(84B)	120.0
C(133)-N(134)-H(13A)	120.0
C(133)-N(134)-H(13B)	120.0
H(13A)-N(134)-H(13B)	120.0
C(182)-N(183)-H(18A)	120.0
C(182)-N(183)-H(18B)	120.0
H(18A)-N(183)-H(18B)	120.0
N(1)-C(1)-C(1A)	107.5(2)

N(1)-C(1)-C(2)	102.5(2)
C(1A)-C(1)-C(2)	113.6(2)
N(1)-C(1)-C(19)	102.6(2)
C(1A)-C(1)-C(19)	109.3(2)
C(2)-C(1)-C(19)	119.8(2)
C(1)-C(1A)-H(1A1)	109.5
C(1)-C(1A)-H(1A2)	109.5
H(1A1)-C(1A)-H(1A2)	109.5
C(1)-C(1A)-H(1A3)	109.5
H(1A1)-C(1A)-H(1A3)	109.5
H(1A2)-C(1A)-H(1A3)	109.5
C(2)-C(2A)-H(2A1)	109.5
C(2)-C(2A)-H(2A2)	109.5
H(2A1)-C(2A)-H(2A2)	109.5
C(2)-C(2A)-H(2A3)	109.5
H(2A1)-C(2A)-H(2A3)	109.5
H(2A2)-C(2A)-H(2A3)	109.5
C(2A)-C(2)-C(21)	110.5(2)
C(2A)-C(2)-C(1)	113.6(2)
C(21)-C(2)-C(1)	109.0(2)
C(2A)-C(2)-C(3)	114.2(2)
C(21)-C(2)-C(3)	106.5(2)
C(1)-C(2)-C(3)	102.5(2)
C(4)-C(3)-C(31)	108.8(2)
C(4)-C(3)-C(2)	101.5(2)
C(31)-C(3)-C(2)	118.7(2)
C(4)-C(3)-H(3)	109.1
C(31)-C(3)-H(3)	109.1
C(2)-C(3)-H(3)	109.1
N(1)-C(4)-C(5)	123.5(3)
N(1)-C(4)-C(3)	112.3(2)
C(5)-C(4)-C(3)	124.2(3)
C(6)-C(5)-C(4)	121.8(3)
C(6)-C(5)-C(51)	122.2(3)
C(4)-C(5)-C(51)	115.9(3)
C(5)-C(6)-N(2)	124.4(3)
C(5)-C(6)-C(7)	127.0(3)
N(2)-C(6)-C(7)	108.5(2)
C(7A)-C(7)-C(6)	116.7(3)

C(7A)-C(7)-C(71)	110.6(3)
C(6)-C(7)-C(71)	105.3(2)
C(7A)-C(7)-C(8)	113.5(2)
C(6)-C(7)-C(8)	100.9(2)
C(71)-C(7)-C(8)	109.0(2)
C(7)-C(7A)-H(7A1)	109.5
C(7)-C(7A)-H(7A2)	109.5
H(7A1)-C(7A)-H(7A2)	109.5
C(7)-C(7A)-H(7A3)	109.5
H(7A1)-C(7A)-H(7A3)	109.5
H(7A2)-C(7A)-H(7A3)	109.5
C(9)-C(8)-C(81)	109.3(2)
C(9)-C(8)-C(7)	102.2(2)
C(81)-C(8)-C(7)	116.8(3)
C(9)-C(8)-H(8)	109.4
C(81)-C(8)-H(8)	109.4
C(7)-C(8)-H(8)	109.4
N(2)-C(9)-C(10)	125.5(3)
N(2)-C(9)-C(8)	111.0(3)
C(10)-C(9)-C(8)	123.3(3)
C(11)-C(10)-C(9)	126.5(3)
C(11)-C(10)-H(10)	116.7
C(9)-C(10)-H(10)	116.7
N(3)-C(11)-C(10)	124.8(3)
N(3)-C(11)-C(12)	110.8(3)
C(10)-C(11)-C(12)	124.3(3)
C(12)-C(12B)-H(12A)	109.5
C(12)-C(12B)-H(12B)	109.5
H(12A)-C(12B)-H(12B)	109.5
C(12)-C(12B)-H(12C)	109.5
H(12A)-C(12B)-H(12C)	109.5
H(12B)-C(12B)-H(12C)	109.5
C(11)-C(12)-C(12A)	114.3(3)
C(11)-C(12)-C(12B)	107.6(2)
C(12A)-C(12)-C(12B)	109.1(3)
C(11)-C(12)-C(13)	99.9(2)
C(12A)-C(12)-C(13)	115.9(2)
C(12B)-C(12)-C(13)	109.4(3)
C(12)-C(12A)-H(12D)	109.5

C(12)-C(12A)-H(12E)	109.5
H(12D)-C(12A)-H(12E)	109.5
C(12)-C(12A)-H(12F)	109.5
H(12D)-C(12A)-H(12F)	109.5
H(12E)-C(12A)-H(12F)	109.5
C(14)-C(13)-C(131)	110.7(2)
C(14)-C(13)-C(12)	101.8(2)
C(131)-C(13)-C(12)	111.8(3)
C(14)-C(13)-H(13)	110.7
C(131)-C(13)-H(13)	110.7
C(12)-C(13)-H(13)	110.7
C(15)-C(14)-N(3)	125.8(3)
C(15)-C(14)-C(13)	126.8(3)
N(3)-C(14)-C(13)	107.3(2)
C(14)-C(15)-C(16)	122.4(3)
C(14)-C(15)-C(151)	118.5(3)
C(16)-C(15)-C(151)	119.2(3)
N(4)-C(16)-C(15)	122.4(3)
N(4)-C(16)-C(17)	111.4(2)
C(15)-C(16)-C(17)	126.2(2)
C(16)-C(17)-C(17B)	107.8(2)
C(16)-C(17)-C(171)	114.5(2)
C(17B)-C(17)-C(171)	111.5(2)
C(16)-C(17)-C(18)	102.2(2)
C(17B)-C(17)-C(18)	112.2(2)
C(171)-C(17)-C(18)	108.3(2)
C(17)-C(17B)-H(17A)	109.5
C(17)-C(17B)-H(17B)	109.5
H(17A)-C(17B)-H(17B)	109.5
C(17)-C(17B)-H(17C)	109.5
H(17A)-C(17B)-H(17C)	109.5
H(17B)-C(17B)-H(17C)	109.5
C(19)-C(18)-C(181)	117.3(2)
C(19)-C(18)-C(17)	104.1(2)
C(181)-C(18)-C(17)	112.5(2)
C(19)-C(18)-H(18)	107.5
C(181)-C(18)-H(18)	107.5
C(17)-C(18)-H(18)	107.5
N(4)-C(19)-C(18)	104.4(2)

N(4)-C(19)-C(1)	104.8(2)
C(18)-C(19)-C(1)	120.8(2)
N(4)-C(19)-H(19)	108.7
C(18)-C(19)-H(19)	108.7
C(1)-C(19)-H(19)	108.7
C(22)-C(21)-C(2)	114.0(2)
C(22)-C(21)-H(21A)	108.8
C(2)-C(21)-H(21A)	108.8
C(22)-C(21)-H(21B)	108.8
C(2)-C(21)-H(21B)	108.8
H(21A)-C(21)-H(21B)	107.7
O(23)-C(22)-N(23)	123.5(3)
O(23)-C(22)-C(21)	121.2(3)
N(23)-C(22)-C(21)	115.3(3)
C(32)-C(31)-C(3)	114.8(2)
C(32)-C(31)-H(31A)	108.6
C(3)-C(31)-H(31A)	108.6
C(32)-C(31)-H(31B)	108.6
C(3)-C(31)-H(31B)	108.6
H(31A)-C(31)-H(31B)	107.5
C(33)-C(32)-C(31)	111.3(3)
C(33)-C(32)-H(32A)	109.4
C(31)-C(32)-H(32A)	109.4
C(33)-C(32)-H(32B)	109.4
C(31)-C(32)-H(32B)	109.4
H(32A)-C(32)-H(32B)	108.0
O(34)-C(33)-N(34)	122.0(4)
O(34)-C(33)-C(32)	122.0(3)
N(34)-C(33)-C(32)	116.0(3)
C(5)-C(51)-H(51A)	109.5
C(5)-C(51)-H(51B)	109.5
H(51A)-C(51)-H(51B)	109.5
C(5)-C(51)-H(51C)	109.5
H(51A)-C(51)-H(51C)	109.5
H(51B)-C(51)-H(51C)	109.5
C(72)-C(71)-C(7)	115.6(3)
C(72)-C(71)-H(71A)	108.4
C(7)-C(71)-H(71A)	108.4
C(72)-C(71)-H(71B)	108.4

C(7)-C(71)-H(71B)	108.4
H(71A)-C(71)-H(71B)	107.4
O(73)-C(72)-N(73)	122.1(3)
O(73)-C(72)-C(71)	121.6(3)
N(73)-C(72)-C(71)	116.3(3)
C(8)-C(81)-C(82)	115.9(3)
C(8)-C(81)-H(81A)	108.3
C(82)-C(81)-H(81A)	108.3
C(8)-C(81)-H(81B)	108.3
C(82)-C(81)-H(81B)	108.3
H(81A)-C(81)-H(81B)	107.4
C(83)-C(82)-C(81)	109.1(3)
C(83)-C(82)-H(82A)	109.9
C(81)-C(82)-H(82A)	109.9
C(83)-C(82)-H(82B)	109.9
C(81)-C(82)-H(82B)	109.9
H(82A)-C(82)-H(82B)	108.3
O(84)-C(83)-N(84)	122.9(3)
O(84)-C(83)-C(82)	120.9(3)
N(84)-C(83)-C(82)	116.2(3)
C(132)-C(131)-C(13)	115.5(3)
C(132)-C(131)-H(13C)	108.4
C(13)-C(131)-H(13C)	108.4
C(132)-C(131)-H(13D)	108.4
C(13)-C(131)-H(13D)	108.4
H(13C)-C(131)-H(13D)	107.5
C(131)-C(132)-C(133)	113.4(3)
C(131)-C(132)-H(13E)	108.9
C(133)-C(132)-H(13E)	108.9
C(131)-C(132)-H(13F)	108.9
C(133)-C(132)-H(13F)	108.9
H(13E)-C(132)-H(13F)	107.7
O(134)-C(133)-N(134)	122.5(3)
O(134)-C(133)-C(132)	121.1(3)
N(134)-C(133)-C(132)	116.5(3)
C(15)-C(151)-H(15A)	109.5
C(15)-C(151)-H(15B)	109.5
H(15A)-C(151)-H(15B)	109.5
C(15)-C(151)-H(15C)	109.5

H(15A)-C(151)-H(15C)	109.5
H(15B)-C(151)-H(15C)	109.5
C(172)-C(171)-C(17)	113.0(2)
C(172)-C(171)-H(17D)	109.0
C(17)-C(171)-H(17D)	109.0
C(172)-C(171)-H(17E)	109.0
C(17)-C(171)-H(17E)	109.0
H(17D)-C(171)-H(17E)	107.8
C(173)-C(172)-C(171)	113.2(2)
C(173)-C(172)-H(17F)	108.9
C(171)-C(172)-H(17F)	108.9
C(173)-C(172)-H(17G)	108.9
C(171)-C(172)-H(17G)	108.9
H(17F)-C(172)-H(17G)	107.8
O(175)-C(173)-O(174)	125.3(3)
O(175)-C(173)-C(172)	117.5(3)
O(174)-C(173)-C(172)	117.1(3)
C(182)-C(181)-C(18)	117.3(3)
C(182)-C(181)-H(18C)	108.0
C(18)-C(181)-H(18C)	108.0
C(182)-C(181)-H(18D)	108.0
C(18)-C(181)-H(18D)	108.0
H(18C)-C(181)-H(18D)	107.2
O(183)-C(182)-N(183)	122.2(3)
O(183)-C(182)-C(181)	121.4(3)
N(183)-C(182)-C(181)	116.4(3)
H(1OA)-O(1)-H(1OB)	104(5)
H(2OB)-O(2)-H(2OA)	107(5)
N(5)-C(23)-C(24)	178.6(6)
C(23)-C(24)-H(24A)	109.5
C(23)-C(24)-H(24B)	109.5
H(24A)-C(24)-H(24B)	109.5
C(23)-C(24)-H(24C)	109.5
H(24A)-C(24)-H(24C)	109.5
H(24B)-C(24)-H(24C)	109.5
N(6)-C(25)-C(26)	178.4(5)
C(25)-C(26)-H(26A)	109.5
C(25)-C(26)-H(26B)	109.5
H(26A)-C(26)-H(26B)	109.5

C(25)-C(26)-H(26C)	109.5
H(26A)-C(26)-H(26C)	109.5
H(26B)-C(26)-H(26C)	109.5

Symmetry transformations used to generate equivalent atoms:

Table S8. Torsion angles [°] for crystalline **Cuby**.

N(3)-Cu-N(1)-C(4)	-132.5(4)
N(4)-Cu-N(1)-C(4)	163.4(3)
N(2)-Cu-N(1)-C(4)	-8.4(3)
N(3)-Cu-N(1)-C(1)	46.1(5)
N(4)-Cu-N(1)-C(1)	-18.00(19)
N(2)-Cu-N(1)-C(1)	170.19(19)
C(4)-N(1)-C(1)-C(1A)	103.8(3)
Cu-N(1)-C(1)-C(1A)	-75.1(2)
C(4)-N(1)-C(1)-C(2)	-16.2(3)
Cu-N(1)-C(1)-C(2)	164.96(17)
C(4)-N(1)-C(1)-C(19)	-141.0(2)
Cu-N(1)-C(1)-C(19)	40.1(2)
N(1)-C(1)-C(2)-C(2A)	149.8(2)
C(1A)-C(1)-C(2)-C(2A)	34.1(3)
C(19)-C(1)-C(2)-C(2A)	-97.6(3)
N(1)-C(1)-C(2)-C(21)	-86.5(3)
C(1A)-C(1)-C(2)-C(21)	157.8(2)
C(19)-C(1)-C(2)-C(21)	26.1(3)
N(1)-C(1)-C(2)-C(3)	26.1(3)
C(1A)-C(1)-C(2)-C(3)	-89.6(3)
C(19)-C(1)-C(2)-C(3)	138.7(2)
C(2A)-C(2)-C(3)-C(4)	-150.0(2)
C(21)-C(2)-C(3)-C(4)	87.7(2)
C(1)-C(2)-C(3)-C(4)	-26.7(3)
C(2A)-C(2)-C(3)-C(31)	-30.9(4)
C(21)-C(2)-C(3)-C(31)	-153.2(2)
C(1)-C(2)-C(3)-C(31)	92.4(3)
C(1)-N(1)-C(4)-C(5)	179.7(3)
Cu-N(1)-C(4)-C(5)	-1.6(4)
C(1)-N(1)-C(4)-C(3)	-1.8(3)
Cu-N(1)-C(4)-C(3)	176.8(2)
C(31)-C(3)-C(4)-N(1)	-107.1(3)
C(2)-C(3)-C(4)-N(1)	18.9(3)
C(31)-C(3)-C(4)-C(5)	71.4(3)
C(2)-C(3)-C(4)-C(5)	-162.7(3)
N(1)-C(4)-C(5)-C(6)	12.4(5)
C(3)-C(4)-C(5)-C(6)	-165.9(3)

N(1)-C(4)-C(5)-C(51)	-165.2(3)
C(3)-C(4)-C(5)-C(51)	16.5(4)
C(4)-C(5)-C(6)-N(2)	-8.3(5)
C(51)-C(5)-C(6)-N(2)	169.2(3)
C(4)-C(5)-C(6)-C(7)	176.6(3)
C(51)-C(5)-C(6)-C(7)	-5.9(5)
C(9)-N(2)-C(6)-C(5)	175.3(3)
Cu-N(2)-C(6)-C(5)	-5.8(4)
C(9)-N(2)-C(6)-C(7)	-8.9(3)
Cu-N(2)-C(6)-C(7)	170.02(18)
C(5)-C(6)-C(7)-C(7A)	-39.7(4)
N(2)-C(6)-C(7)-C(7A)	144.6(3)
C(5)-C(6)-C(7)-C(71)	83.5(4)
N(2)-C(6)-C(7)-C(71)	-92.2(3)
C(5)-C(6)-C(7)-C(8)	-163.2(3)
N(2)-C(6)-C(7)-C(8)	21.1(3)
C(7A)-C(7)-C(8)-C(9)	-149.8(2)
C(6)-C(7)-C(8)-C(9)	-24.1(3)
C(71)-C(7)-C(8)-C(9)	86.4(3)
C(7A)-C(7)-C(8)-C(81)	-30.7(4)
C(6)-C(7)-C(8)-C(81)	95.0(3)
C(71)-C(7)-C(8)-C(81)	-154.4(2)
C(6)-N(2)-C(9)-C(10)	175.8(3)
Cu-N(2)-C(9)-C(10)	-3.2(4)
C(6)-N(2)-C(9)-C(8)	-8.4(3)
Cu-N(2)-C(9)-C(8)	172.63(18)
C(81)-C(8)-C(9)-N(2)	-103.0(3)
C(7)-C(8)-C(9)-N(2)	21.4(3)
C(81)-C(8)-C(9)-C(10)	73.0(4)
C(7)-C(8)-C(9)-C(10)	-162.7(3)
N(2)-C(9)-C(10)-C(11)	4.7(5)
C(8)-C(9)-C(10)-C(11)	-170.6(3)
C(14)-N(3)-C(11)-C(10)	176.1(3)
Cu-N(3)-C(11)-C(10)	-3.1(4)
C(14)-N(3)-C(11)-C(12)	-6.9(3)
Cu-N(3)-C(11)-C(12)	173.84(19)
C(9)-C(10)-C(11)-N(3)	-1.2(5)
C(9)-C(10)-C(11)-C(12)	-177.7(3)
N(3)-C(11)-C(12)-C(12A)	148.2(3)

C(10)-C(11)-C(12)-C(12A)	-34.8(4)
N(3)-C(11)-C(12)-C(12B)	-90.4(3)
C(10)-C(11)-C(12)-C(12B)	86.6(4)
N(3)-C(11)-C(12)-C(13)	23.8(3)
C(10)-C(11)-C(12)-C(13)	-159.3(3)
C(11)-C(12)-C(13)-C(14)	-29.4(3)
C(12A)-C(12)-C(13)-C(14)	-152.8(3)
C(12B)-C(12)-C(13)-C(14)	83.3(3)
C(11)-C(12)-C(13)-C(131)	88.8(3)
C(12A)-C(12)-C(13)-C(131)	-34.6(4)
C(12B)-C(12)-C(13)-C(131)	-158.4(2)
C(11)-N(3)-C(14)-C(15)	168.3(3)
Cu-N(3)-C(14)-C(15)	-12.5(4)
C(11)-N(3)-C(14)-C(13)	-13.9(3)
Cu-N(3)-C(14)-C(13)	165.30(19)
C(131)-C(13)-C(14)-C(15)	86.7(4)
C(12)-C(13)-C(14)-C(15)	-154.4(3)
C(131)-C(13)-C(14)-N(3)	-91.1(3)
C(12)-C(13)-C(14)-N(3)	27.9(3)
N(3)-C(14)-C(15)-C(16)	6.7(4)
C(13)-C(14)-C(15)-C(16)	-170.6(3)
N(3)-C(14)-C(15)-C(151)	-174.6(3)
C(13)-C(14)-C(15)-C(151)	8.1(4)
C(19)-N(4)-C(16)-C(15)	177.1(2)
Cu-N(4)-C(16)-C(15)	5.7(4)
C(19)-N(4)-C(16)-C(17)	-3.9(3)
Cu-N(4)-C(16)-C(17)	-175.25(18)
C(14)-C(15)-C(16)-N(4)	-3.0(4)
C(151)-C(15)-C(16)-N(4)	178.3(3)
C(14)-C(15)-C(16)-C(17)	178.1(3)
C(151)-C(15)-C(16)-C(17)	-0.6(4)
N(4)-C(16)-C(17)-C(17B)	-103.5(3)
C(15)-C(16)-C(17)-C(17B)	75.5(3)
N(4)-C(16)-C(17)-C(171)	131.8(3)
C(15)-C(16)-C(17)-C(171)	-49.2(4)
N(4)-C(16)-C(17)-C(18)	15.0(3)
C(15)-C(16)-C(17)-C(18)	-166.0(3)
C(16)-C(17)-C(18)-C(19)	-19.4(3)
C(17B)-C(17)-C(18)-C(19)	95.9(3)

C(171)-C(17)-C(18)-C(19)	-140.6(2)
C(16)-C(17)-C(18)-C(181)	-147.4(2)
C(17B)-C(17)-C(18)-C(181)	-32.1(3)
C(171)-C(17)-C(18)-C(181)	91.4(3)
C(16)-N(4)-C(19)-C(18)	-9.4(3)
Cu-N(4)-C(19)-C(18)	163.45(17)
C(16)-N(4)-C(19)-C(1)	-137.3(2)
Cu-N(4)-C(19)-C(1)	35.5(3)
C(181)-C(18)-C(19)-N(4)	142.7(2)
C(17)-C(18)-C(19)-N(4)	17.7(3)
C(181)-C(18)-C(19)-C(1)	-99.9(3)
C(17)-C(18)-C(19)-C(1)	135.1(2)
N(1)-C(1)-C(19)-N(4)	-45.0(3)
C(1A)-C(1)-C(19)-N(4)	68.9(3)
C(2)-C(1)-C(19)-N(4)	-157.6(2)
N(1)-C(1)-C(19)-C(18)	-162.2(2)
C(1A)-C(1)-C(19)-C(18)	-48.3(3)
C(2)-C(1)-C(19)-C(18)	85.2(3)
C(2A)-C(2)-C(21)-C(22)	-57.6(3)
C(1)-C(2)-C(21)-C(22)	176.8(2)
C(3)-C(2)-C(21)-C(22)	66.9(3)
C(2)-C(21)-C(22)-O(23)	-80.3(4)
C(2)-C(21)-C(22)-N(23)	99.7(3)
C(4)-C(3)-C(31)-C(32)	-155.8(3)
C(2)-C(3)-C(31)-C(32)	88.9(3)
C(3)-C(31)-C(32)-C(33)	69.4(3)
C(31)-C(32)-C(33)-O(34)	58.0(4)
C(31)-C(32)-C(33)-N(34)	-120.7(3)
C(7A)-C(7)-C(71)-C(72)	-50.5(4)
C(6)-C(7)-C(71)-C(72)	-177.4(3)
C(8)-C(7)-C(71)-C(72)	75.0(3)
C(7)-C(71)-C(72)-O(73)	-35.3(5)
C(7)-C(71)-C(72)-N(73)	145.3(3)
C(9)-C(8)-C(81)-C(82)	51.0(4)
C(7)-C(8)-C(81)-C(82)	-64.3(4)
C(8)-C(81)-C(82)-C(83)	-170.4(3)
C(81)-C(82)-C(83)-O(84)	100.4(4)
C(81)-C(82)-C(83)-N(84)	-77.3(4)
C(14)-C(13)-C(131)-C(132)	-67.4(3)

C(12)-C(13)-C(131)-C(132)	179.9(3)
C(13)-C(131)-C(132)-C(133)	-179.1(3)
C(131)-C(132)-C(133)-O(134)	26.4(4)
C(131)-C(132)-C(133)-N(134)	-153.3(3)
C(16)-C(17)-C(171)-C(172)	-44.5(3)
C(17B)-C(17)-C(171)-C(172)	-167.3(3)
C(18)-C(17)-C(171)-C(172)	68.8(3)
C(17)-C(171)-C(172)-C(173)	175.4(3)
C(171)-C(172)-C(173)-O(175)	64.4(4)
C(171)-C(172)-C(173)-O(174)	-116.6(3)
C(19)-C(18)-C(181)-C(182)	89.1(3)
C(17)-C(18)-C(181)-C(182)	-150.2(3)
C(18)-C(181)-C(182)-O(183)	-118.3(3)
C(18)-C(181)-C(182)-N(183)	63.4(4)

Symmetry transformations used to generate equivalent atoms:

#1 -x,y+1/2,-z+1 #2 -x,y+1/2,-z #3 x+1,y,z-1

#4 x+1,y,z #5 -x,y-1/2,-z #6 x,y,z-1 #7 -x,y-1/2,-z+1

#8 x-1,y,z+1 #9 x,y,z+1

Table S9. Anisotropic displacement parameters ($Å^2$x 10^3) for crystalline **Cuby**. The anisotropic displacement factor exponent takes the form: $-2\pi^2[h^2 a^{*2}U^{11} + ... + 2 h k a^* b^* U^{12}]$

	U^{11}	U^{22}	U^{33}	U^{23}	U^{13}	U^{12}
Cu	21(1)	15(1)	15(1)	-1(1)	3(1)	3(1)
N(1)	22(1)	15(1)	16(1)	-3(1)	3(1)	2(1)
N(2)	19(1)	16(1)	18(1)	-2(1)	5(1)	1(1)
N(3)	19(1)	16(1)	19(1)	-1(1)	4(1)	1(1)
N(4)	17(1)	13(1)	17(1)	-1(1)	4(1)	2(1)
N(23)	44(2)	16(1)	28(2)	2(1)	9(1)	5(1)
N(34)	91(3)	26(2)	34(2)	1(1)	1(2)	-8(2)
N(73)	44(2)	35(2)	29(2)	-3(1)	18(1)	-5(1)
N(84)	44(2)	29(2)	37(2)	-1(1)	-4(1)	-13(1)
N(134)	28(2)	28(2)	38(2)	-3(1)	15(1)	-6(1)
N(183)	27(2)	21(1)	30(2)	4(1)	11(1)	7(1)
O(23)	46(2)	23(1)	25(1)	5(1)	1(1)	4(1)
O(34)	56(2)	30(1)	29(1)	1(1)	0(1)	0(1)
O(73)	104(3)	32(2)	79(2)	-26(2)	72(2)	-26(2)
O(84)	65(2)	33(1)	30(1)	2(1)	13(1)	-14(1)
O(134)	34(1)	24(1)	40(1)	-7(1)	15(1)	-9(1)
O(174)	47(1)	20(1)	37(1)	6(1)	18(1)	4(1)
O(175)	34(1)	26(1)	25(1)	1(1)	14(1)	2(1)
O(183)	33(1)	19(1)	24(1)	0(1)	4(1)	3(1)
C(1)	18(1)	16(1)	14(1)	-2(1)	3(1)	1(1)
C(1A)	22(2)	18(2)	22(2)	1(1)	1(1)	-4(1)
C(2A)	20(2)	21(2)	22(2)	-4(1)	4(1)	0(1)
C(2)	17(1)	16(1)	16(1)	0(1)	1(1)	0(1)
C(3)	20(1)	14(1)	18(2)	-1(1)	1(1)	2(1)
C(4)	18(1)	13(1)	18(2)	-2(1)	2(1)	-2(1)
C(5)	23(2)	19(2)	16(1)	-1(1)	2(1)	0(1)
C(6)	19(1)	18(2)	16(1)	-1(1)	3(1)	-4(1)
C(7)	24(2)	19(2)	18(1)	-2(1)	7(1)	-2(1)
C(7A)	36(2)	23(2)	22(2)	-3(1)	2(1)	-1(1)
C(8)	23(2)	18(2)	19(2)	-4(1)	8(1)	-1(1)
C(9)	18(1)	19(2)	21(2)	-4(1)	8(1)	-2(1)
C(10)	22(2)	17(2)	27(2)	-4(1)	8(1)	5(1)
C(11)	16(1)	18(2)	25(2)	1(1)	7(1)	0(1)
C(12B)	21(2)	43(2)	38(2)	12(2)	10(2)	8(2)

C(12)	21(2)	17(2)	28(2)	6(1)	8(1)	6(1)
C(12A)	50(2)	20(2)	33(2)	5(1)	22(2)	12(2)
C(13)	18(1)	17(2)	24(2)	5(1)	4(1)	6(1)
C(14)	13(1)	18(1)	22(2)	4(1)	2(1)	1(1)
C(15)	13(1)	18(1)	21(2)	2(1)	3(1)	-1(1)
C(16)	10(1)	20(2)	18(1)	0(1)	3(1)	-3(1)
C(17)	16(1)	17(1)	17(1)	-2(1)	1(1)	-1(1)
C(17B)	18(2)	22(2)	27(2)	-3(1)	-2(1)	-2(1)
C(18)	16(1)	17(1)	16(1)	-1(1)	2(1)	0(1)
C(19)	15(1)	13(1)	18(1)	-1(1)	3(1)	1(1)
C(21)	19(2)	15(1)	20(2)	0(1)	3(1)	1(1)
C(22)	21(2)	17(2)	29(2)	3(1)	3(1)	-2(1)
C(31)	24(2)	20(2)	25(2)	1(1)	-1(1)	-1(1)
C(32)	21(2)	27(2)	32(2)	-1(1)	-1(1)	-1(1)
C(33)	26(2)	24(2)	31(2)	2(1)	-8(1)	1(1)
C(51)	40(2)	32(2)	18(2)	2(1)	6(1)	9(2)
C(71)	32(2)	21(2)	27(2)	-7(1)	10(1)	-5(1)
C(72)	30(2)	31(2)	33(2)	-9(2)	14(1)	-8(2)
C(81)	28(2)	21(2)	22(2)	-5(1)	8(1)	-4(1)
C(82)	32(2)	23(2)	26(2)	-8(1)	10(1)	-4(1)
C(83)	28(2)	24(2)	25(2)	-3(1)	13(1)	-2(1)
C(131)	22(2)	15(2)	28(2)	2(1)	6(1)	0(1)
C(132)	26(2)	19(2)	28(2)	-1(1)	6(1)	-2(1)
C(133)	26(2)	24(2)	18(2)	3(1)	4(1)	0(1)
C(151)	26(2)	22(2)	23(2)	0(1)	0(1)	4(1)
C(171)	19(1)	22(2)	14(1)	-1(1)	1(1)	2(1)
C(172)	23(1)	27(2)	17(1)	2(2)	5(1)	-4(2)
C(173)	18(2)	26(2)	22(2)	1(1)	4(1)	1(1)
C(181)	22(2)	20(2)	20(2)	-6(1)	3(1)	1(1)
C(182)	25(2)	19(2)	21(2)	-10(1)	-2(1)	2(1)
O(1)	38(2)	31(2)	42(2)	4(1)	8(1)	2(1)
O(2)	53(2)	22(1)	42(2)	3(1)	19(1)	-6(1)
N(5)	82(4)	85(4)	108(5)	-23(3)	-20(3)	-18(3)
C(23)	69(3)	54(3)	45(3)	-10(2)	-10(2)	4(3)
C(24)	70(3)	60(3)	47(3)	7(2)	6(2)	-2(2)
N(6)	76(3)	45(2)	86(3)	19(2)	-6(2)	-17(2)
C(25)	41(2)	39(2)	48(2)	-5(2)	-10(2)	3(2)
C(26)	33(2)	35(2)	49(3)	-2(2)	8(2)	1(2)

Table S10. Hydrogen coordinates (x 10^4) & isotropic displacement parameters (Å^2 x 10^3) for **Cuby**.

	x	y	z	U(eq)
H(23A)	1256	7501	2464	35
H(23B)	1731	7131	3251	35
H(34A)	-1633	6800	-379	61
H(34B)	-2058	6818	583	61
H(73A)	8618	4700	-2164	42
H(73B)	8110	5116	-1484	42
H(84A)	-1275	2495	-482	45
H(84B)	-532	3033	-807	45
H(13A)	310	2397	5551	37
H(13B)	1442	2913	5774	37
H(18A)	-303	6464	5414	31
H(18B)	784	5957	5730	31
H(1A1)	71	5076	3840	31
H(1A2)	-357	4833	2871	31
H(1A3)	1263	4567	3535	31
H(2A1)	-1478	5741	2979	32
H(2A2)	-144	6126	3611	32
H(2A3)	-1034	6347	2679	32
H(3)	1103	5965	1196	21
H(7A1)	3639	4109	-1534	41
H(7A2)	1930	4098	-909	41
H(7A3)	2777	4665	-1203	41
H(8)	6221	3570	-152	23
H(10)	7060	3125	1357	26
H(12A)	10764	2980	3555	50
H(12B)	10440	3253	2603	50
H(12C)	10046	3595	3460	50
H(12D)	8598	2218	3158	50
H(12E)	6494	2325	2709	50
H(12F)	8304	2446	2174	50
H(13)	7997	3073	4501	24
H(17A)	6746	5549	5961	34
H(17B)	7811	5000	5734	34
H(17C)	7456	5462	5004	34
H(18)	2275	5329	4782	20
H(19)	4786	5614	3503	18

H(21A)	3231	6351	3212	21
H(21B)	3898	6131	2303	21
H(31A)	-1023	5160	790	27
H(31B)	-1652	5217	1767	27
H(32A)	-3928	5676	875	32
H(32B)	-2700	6136	1404	32
H(51A)	3298	5319	-565	44
H(51B)	2393	5756	53	44
H(51C)	1095	5280	-389	44
H(71A)	6119	4970	-466	31
H(71B)	7346	4520	79	31
H(81A)	4031	2908	152	28
H(81B)	3087	3241	-670	28
H(82A)	2050	3436	1083	32
H(82B)	962	3673	199	32
H(13C)	4310	2833	3589	25
H(13D)	5678	2375	4031	25
H(13E)	3886	3195	4986	29
H(13F)	5279	2743	5434	29
H(15A)	5746	3780	5832	35
H(15B)	7506	4191	5850	35
H(15C)	7731	3584	5504	35
H(17D)	4941	4581	6352	22
H(17E)	3547	5094	6319	22
H(17F)	1329	4639	5332	27
H(17G)	2748	4135	5285	27
H(18C)	4387	6008	5653	25
H(18D)	4896	6199	4703	25
H(1OA)	650(80)	5234(11)	6970(40)	80(20)
H(1OB)	1690(50)	5650(20)	7270(30)	62(16)
H(2OB)	-1050(50)	3483(15)	8000(30)	46(13)
H(2OA)	60(70)	3370(20)	7370(20)	63(16)
H(24A)	8661	6338	7771	89
H(24B)	7509	6884	7928	89
H(24C)	7696	6670	6950	89
H(26A)	3750	3254	7416	58
H(26B)	5416	2885	7086	58
H(26C)	5585	3116	8069	58

Table S11. Hydrogen bonds for crystalline **Cuby** [Å and °].

D-H...A	d(D-H)	d(H...A)	d(D...A)	<(DHA)
N(23)-H(23A)...O(2)#1	0.88	2.02	2.896(4)	171.0
N(23)-H(23B)...O(183)	0.88	2.07	2.928(4)	166.5
N(34)-H(34A)...O(84)#2	0.88	2.21	2.994(4)	148.2
N(34)-H(34B)...N(6)#1	0.88	2.27	3.123(6)	164.3
N(73)-H(73A)...O(175)#3	0.88	2.12	2.974(4)	161.9
N(73)-H(73B)...O(34)#4	0.88	2.24	3.088(4)	160.7
N(84)-H(84A)...O(23)#5	0.88	2.12	2.889(4)	146.3
N(84)-H(84B)...O(2)#6	0.88	2.16	2.966(4)	151.3
N(134)-H(13A)...O(183)#7	0.88	2.22	3.093(4)	170.8
N(134)-H(13B)...O(174)	0.88	2.11	2.987(4)	174.8
N(183)-H(18A)...O(134)#1	0.88	2.06	2.933(4)	175.3
N(183)-H(18B)...O(1)	0.88	2.10	2.893(4)	149.0
C(3)-H(3)...O(23)	1.00	2.28	3.096(4)	138.6
C(8)-H(8)...O(73)	1.00	2.40	3.047(4)	122.0
O(1)-H(1OA)...O(175)	0.83(3)	1.91(3)	2.738(4)	177(6)
O(1)-H(1OB)...N(5)	0.84(3)	2.15(3)	2.950(6)	157(5)
O(2)-H(2OB)...O(73)#8	0.82(3)	1.87(3)	2.688(4)	173(4)
O(2)-H(2OA)...O(174)	0.82(3)	1.88(3)	2.686(3)	165(5)
C(26)-H(26A)...O(174)	0.98	2.25	3.162(5)	154.1
C(26)-H(26C)...O(73)#9	0.98	2.36	3.169(5)	139.4

Symmetry transformations used to generate equivalent atoms:

#1 -x,y+1/2,-z+1 #2 -x,y+1/2,-z #3 x+1,y,z-1

#4 x+1,y,z #5 -x,y-1/2,-z #6 x,y,z-1 #7 -x,y-1/2,-z+1

#8 x-1,y,z+1 #9 x,y,z+1

10. References

1. C. Kieninger, E. Deery, A. D. Lawrence, M. Podewitz, K. Wurst, E. Nemoto-Smith, F. J. Widner, J. A. Baker, S. Jockusch, C. R. Kreutz, K. R. Liedl, K. Gruber, M. J. Warren and B. Kräutler, *Angew. Chem. Int. Ed.* , 2019, **58**, 10756-10760.
2. G. Schlingmann and V. B. Koppenhagen, Zürich (Switzerland), 1979.
3. G. Schlingmann, B. Dresow, L. Ernst and V. B. Koppenhagen, *Liebigs Annalen der Chemie*, 1981, 2061-2066.
4. C. Kieninger, J. A. Baker, M. Podewitz, K. Wurst, S. Jockusch, A. D. Lawrence, E. Deery, K. Gruber, K. R. Liedl, M. J. Warren and B. Kräutler, *Angew. Chem. Int. Ed.*, 2019, **58**, 14568-14572.
5. H.-J. Werner, P. J. Knowles, F. R. Manby, J. A. Black, K. Doll, A. Heßelmann, D. Kats, A. Köhn, T. Korona, D. A. Kreplin, Q. Ma, T. F. Miller, III, A. Mitrushchenkov, K. A. Peterson, I. Polyak, G. Rauhut and M. Sibaev, *J. Chem. Phys.*, 2020, **152**, 144107.
6. K. A. Peterson, D. E. Woon and T. H. Dunning, Jr., *J. Chem. Phys.*, 1994, **100**, 7410-7415.
7. N. B. Balabanov and K. A. Peterson, *J. Chem. Phys.*, 2005, **123**, 064107.
8. N. B. Balabanov and K. A. Peterson, *J. Chem. Phys.*, 2006, **125**, 074110.
9. G. Knizia, *J. Chem. Theory Computation*, 2013, **9**, 4834-4843.
10. C. Kieninger, K. Wurst, M. Podewitz, M. Stanley, E. Deery, A. D. Lawrence, K. R. Liedl, M. J. Warren and B. Kräutler, *Angew. Chem. Int. Ed.*, 2020, **59**, 20129-20136.
11. G. M. Sheldrick, *Acta Cryst. A - Foundation & Advances*, 2015, **71A**, 3-8.
12. G. M. Sheldrick, *Acta Cryst. C-Structural Chemistry*, 2015, **71C**, 3-8.
13. V. B. Pett, M. N. Liebman, P. Murray-Rust, K. Prasad and J. P. Glusker, *J. Am. Chem. Soc.*, 1987, **109**, 3207-3215.
